# Organocatalytic asymmetric allylic amination of Morita–Baylis–Hillman carbonates of isatins

**DOI:** 10.3762/bjoc.8.139

**Published:** 2012-08-06

**Authors:** Hang Zhang, Shan-Jun Zhang, Qing-Qing Zhou, Lin Dong, Ying-Chun Chen

**Affiliations:** 1 Key Laboratory of Drug-Targeting and Drug Delivery System of the Education Ministry, Department of Medicinal Chemistry, West China School of Pharmacy, Sichuan University, Chengdu 610041, China

**Keywords:** allylic amination, asymmetric organocatalysis, Morita–Baylis–Hillman carbonates, 2-oxindoles, quaternary chiral center

## Abstract

The investigation of a Lewis base catalyzed asymmetric allylic amination of Morita–Baylis–Hillman carbonates derived from isatins afforded an electrophilic pathway to access multifunctional oxindoles bearing a C3-quaternary stereocenter, provided with good to excellent enantioselectivity (up to 94% ee) and in high yields (up to 97%).

## Introduction

Chiral 3-amino-2-oxindoles are versatile and useful units for the preparation of natural products and drug candidates, such as the vasopressin VIb receptor antagonist SSR-149415 [1–2], the potent gastrin/CCK-B receptor antagonist AG-041R [3], chartelline C [4–5] and psychotrimine [6]. Therefore, the development of asymmetric protocols to construct such chiral scaffolds has provoked wide interest. The application of 3-substituted oxindoles as nucleophiles in the reactions with azodicarboxylates or nitrosobenzene provides a very simple and direct approach for the synthesis of optically active 3-amino-2-oxindole derivatives [7], either by the catalysis of chiral metal complexes [8–10] or organic catalysts [11–15]. On the other hand, the asymmetric addition to electrophilic imines of isatins is also an attractive pathway, and a variety of examples have been presented [16–21].

**Scheme 1:**
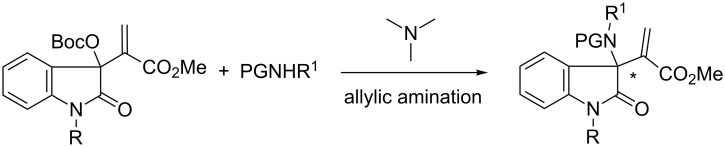
Allylic amination of MBH carbonates of isatins to access 3-amino-2-oxindoles.

Recently, we have developed the asymmetric allylic alkylation reactions [22] with Morita–Baylis–Hillman (MBH) carbonates of isatins to obtain 2-oxindoles bearing a C3-quaternary chiral center, by the catalysis of chiral tertiary amines, β-isocupreidine (β-ICD) or its derivatives [23–24]. We envisaged that such a catalytic strategy should be applicable to the allylic amination of the corresponding MBH carbonates [25–28], as outlined in Scheme 1. Thus, multifunctional chiral 3-amino-2-oxindoles could be obtained in a straightforward manner.

## Results and Discussion

Based on the above considerations, we initially investigated the reaction of MBH carbonate **2a** and a diversity of nucleophilic nitrogen sources by the catalysis of DABCO. No desired reaction occurred for phthalimide [25] or *N*-allyl *p*-toluenesulfonamide [27], which has been successfully applied in the asymmetric amination of MBH carbonates derived from aryl aldehydes. Pleasingly, the reaction took place smoothly to afford product **4a** when hydroxylamine **3a** with *N*-benzyloxycarbonyl and *O*-benzyl groups [29] was applied in diethyl ether (Table 1, entry 1). Subsequently, an array of tertiary amines derived from quinidine was explored to introduce chirality into the product. While poor enantioselectivity was obtained when β-isocupreidine **1a** (β-ICD) or β-isoquinidine **1b** was used (Table 1, entries 2 and 3), a moderate ee value was attained in the presence of *O*-MOM isocupreidine **1c** (Table 1, entry 4) [30]. Moreover, even a slightly higher enantiocontrol was observed for a phenyl-substituted amine **1d** (Table 1, entry 5) [31]. Consequently, we paid attention to the structural modifications on the nitrogen source. Very poor conversion was observed when *N*-Boc protected hydroxylamine **3b** was applied in the catalysis by **1d** (Table 1, entry 6). To our gratification, dramatically improved enantioselectivity was obtained for **3c** bearing an *O*-TES group (Table 1, entry 7), and an even higher ee value was gained for **3d** with a bulkier *O*-TBS group, although it exhibited lower reactivity (Table 1, entry 8). Nevertheless, inferior results were afforded for **3e** with an *O*-TIPS group (Table 1, entry 9). Furthermore, we prepared more isocupreidines with diverse aryl-substitutions and tested their catalytic efficacy in the reaction of MBH carbonate **2a** and **3d**. Diminished enantioselectivity was delivered in the presence of catalysts **1e**–**1g** (Table 1, entries 10–12), but a slightly higher ee value could be obtained upon catalysis by **1h** with a 4-*tert*-butylphenyl group (Table 1, entry 13). In addition, a number of solvents were investigated in the catalysis by **1d** (Table 1, entries 14–18), and chlorobenzene was found to be the optimal selection (Table 1, entry 18). Finally, it was found that the reaction still proceeded smoothly at 0 °C, and a high ee value could be obtained in the catalysis by amine **1h**, although a longer reaction time was required to give a better yield (Table 1, entry 19). It should be noted that the reaction became sluggish when (*S*)-BINOL [32] was added, and even no reaction happened in the presence of other additives such as LiClO_4_ or Ti(OiPr)_4_ [33].

**Table 1:** Screening studies of asymmetric allylic amination of MBH carbonate of isatin.^a^

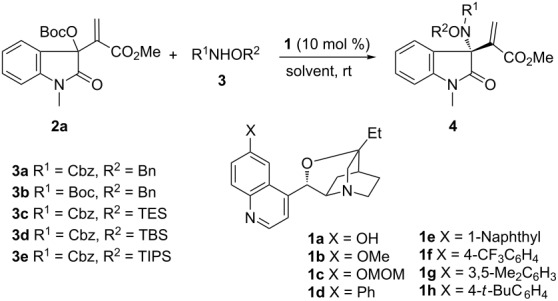						

entry	**1**	**3**	solvent	*t* (h)	yield^b^ (%)	ee^c^ (%)

1	DABCO	**3a**	Et_2_O	12	**4a**, 86	–
2	**1a**	**3a**	Et_2_O	12	**4a**, 83	17
3	**1b**	**3a**	Et_2_O	12	**4a**, 80	37
4	**1c**	**3a**	Et_2_O	12	**4a**, 85	52
5	**1d**	**3a**	Et_2_O	12	**4a**, 85	58
6	**1d**	**3b**	Et_2_O	12	**4b**, <10	–
7	**1d**	**3c**	Et_2_O	12	**4c**, 80	72
8	**1d**	**3d**	Et_2_O	24	**4d**, 85	83
9	**1d**	**3e**	Et_2_O	12	**4e**, 53	72
10	**1e**	**3d**	Et_2_O	24	**4d**, 88	77
11	**1f**	**3d**	Et_2_O	24	**4d**, 78	72
12	**1g**	**3d**	Et_2_O	24	**4d**, 90	74
13	**1h**	**3d**	Et_2_O	24	**4d**, 88	85
14	**1d**	**3d**	DCE	18	**4d**, 74	74
15	**1d**	**3d**	PhCF_3_	12	**4d**, 93	86
16	**1d**	**3d**	*m*-xylene	12	**4d**, 97	82
17	**1d**	**3d**	PhF	12	**4d**, 83	85
18	**1d**	**3d**	PhCl	12	**4d**, 93	88
19^d^	**1h**	**3d**	**PhCl**	**24**	**4d, 92**	**91**

^a^Unless otherwise noted, reactions were performed with 0.12 mmol of **2a**, 0.1 mmol of **3**, and 0.01 mmol **1** of in 0.5 mL solvent at room temperature. ^b^Isolated yield. ^c^Based on chiral HPLC analysis. ^d^At 0 °C.

With the optimized conditions in hand, we explored a diversity of MBH carbonates derived from isatins in the reactions with protected hydroxylamine **3d** by the catalysis of chiral amine **1h** in chlorobenzene at 0 °C. The results are summarized in Table 2. A series of MBH carbonates **2** bearing either electron-donating or -withdrawing substituents on the aromatic moiety of 2-oxindoles were well tolerated. A higher reactivity was generally observed for MBH carbonates with electron-donating substitutions, and excellent yields and enantioselectivity were obtained (Table 2, entries 2–4). On the other hand, MBH carbonates with electron-withdrawing groups exhibited a slower reaction rate, but both good yields and ee values were still obtained (Table 2, entries 5–10).

**Table 2:** Substrate scope and limitations.^a^

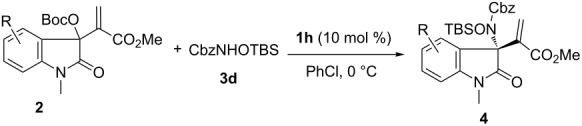				

entry	R	*t* (h)	yield^b^ (%)	ee^c^ (%)

1	H (**2a**)	24	**4d**, 92	91
2	5-Me (**2b**)	24	**4f**, 93	91
3	5-MeO (**2c**)	26	**4g**, 97	94
4	5,7-Me_2_ (**2d**)	40	**4h**, 93	90
5	5-F (**2e**)	36	**4i**, 86	90
6	5-Cl (**2f**)	30	**4j**, 88	89
7	5-Br (**2g**)	36	**4k**, 81	88
8	5-I (**2h**)	36	**4l**, 85	86
9	5-CF_3_O (**2i**)	36	**4m**, 71	85
10	7-F (**2j**)	36	**4n**, 85	90

^a^Reactions were performed with 0.12 mmol of **2**, 0.1 mmol of **3d**, and 0.01 mmol of **1h** in 0.5 mL of chlorobenzene at 0 °C. ^b^Isolated yield. ^c^Based on chiral HPLC analysis.

**Scheme 2:**
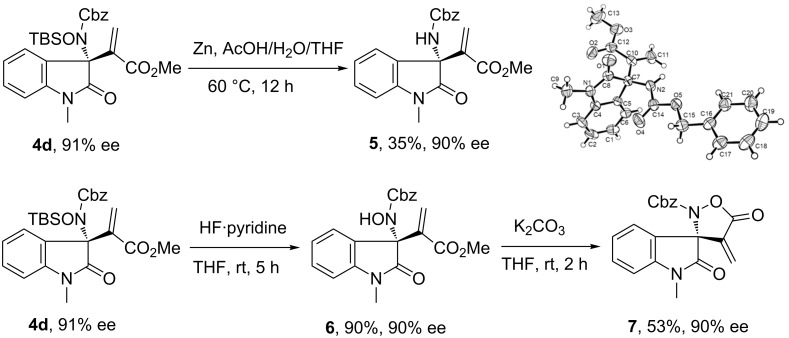
Synthetic transformations of multifunctional product **4d**.

As outlined in Scheme 2, some synthetic transformations were conducted with the multifunctional allylic amination product **4d**. The N–O bond cleavage of **4d** could be realized with Zn powder in acetic acid to produce compound **5**, albeit in modest yield [34–35], whose absolute configuration has been determined by X-ray analysis [36]. The removal of the *O*-TBS unit proceeded efficiently in the presence of hydrofluoric acid, and an intramolecular transesterification process of intermediate **6** happened to afford a spirocyclic oxindole **7** without loss of enantiopurity [37].

## Conclusion

We have developed a highly enantioselective allylic amination of Morita–Baylis–Hillman carbonates of isatins with *N*-silyloxycarbamates by the catalysis of a modified β-ICD derivative, which provides an electrophilic process to 3-amino-2-oxindoles with a C3-quaternary chiral center. A range of products with high molecular complexity were obtained with good to excellent enantioselectivity (up to 94% ee) and high yields (up to 97%). Currently, more studies on the catalytic asymmetric transformations of MBH carbonates of isatins are under way in our laboratory.

## Supporting Information

General experimental procedures, copies of ^1^H, ^13^C NMR spectra and HPLC chromatograms for all new compounds, crystal data and structure refinement for enantiopure **5**.

File 1 General procedures and analytical data.
