# Supplementary material for: Organocatalytic asymmetric allylic amination of Morita–Baylis–Hillman carbonates of isatins
Source: Beilstein J Org Chem. 2012 Aug 6;8:1241–5. doi: 10.3762/bjoc.8.139 (PMC3458744; doi:10.3762/bjoc.8.139)
Supplement: File 1 — General procedures and analytical data. [file Beilstein_J_Org_Chem-08-1241-s001.pdf]

Supporting Information

for

Organocatalytic asymmetric allylic amination of Morita–Baylis–Hillman carbonates of isatins

Hang Zhang, Shan-Jun Zhang, Qing-Qing Zhou, Lin Dong, and Ying-Chun Chen*

Address: Key Laboratory of Drug-Targeting and Drug Delivery System of the Education Ministry, Department of Medicinal Chemistry, West China School of Pharmacy, Sichuan University, Chengdu 610041, China

Email: Ying-Chun Chen* - ycchenhuaxi@yahoo.com.cn

*Corresponding author

General procedures and analytical data

Table of Contents

1. General methods ……………………………………………………………………………S2

2. Preparation of modified β-ICD-type catalysts …………………………………………….. S2

3. General procedure for assembly of MBH carbonates and *N*-silyloxycarbamates ……………..S3

4. Synthetic transformations of multifunctional adduct **4d** ………………………………………S7

5. Crystal data and structure refinement for enantiopure **5** ………………………………………S9

6. NMR spectra and HPLC chromatograms …………………………………………………….S11

7. References…………………………………………………………………………………..S39

1. General Methods

NMR spectra were recorded with tetramethylsilane as the internal standard. TLC was performed on glass-backed silica plates. Column chromatography was performed using silica gel (200–300 mesh) eluting with ethyl acetate and petroleum ether (PE) (EtOAc/PE). ^{1}H NMR spectra were recorded at 400 MHz (Varian) and ^{13}C NMR spectra were recorded at 100 MHz (Varian). Chemical shifts are reported in ppm downfield from $CDCl_3$ (δ = 7.27 ppm) for ^{1}H NMR and relative to the central $CDCl_3$ resonance (δ = 77.0 ppm) for ^{13}C NMR spectroscopy. Coupling constants are given in hertz (Hz). Optical rotations were measured at 589 nm at 20 °C. Enantiomeric excess was determined by HPLC analysis on Chiralpak IC and Chiralcel OD columns. Et_2O was distilled from sodium (Na) under an argon (Ar) atmosphere. Mesitylene was distilled from CaH_2. Chlorobenzene was dried by 4 Å. All other chemicals were used as commercially available, without purification. Cinchona alkaloids catalysts **1a**, **1b**, **1d** and **1e**–**1h** were prepared according to the literature procedure [1]. Catalysts **1c** was prepared according to the literature procedure [2]. Morita−Baylis−Hillman carbonates of isatins [3] and N-silyloxycarbamates were prepared according to the literature procedure [4].

2. Preparation of modified β-ICD-type catalysts

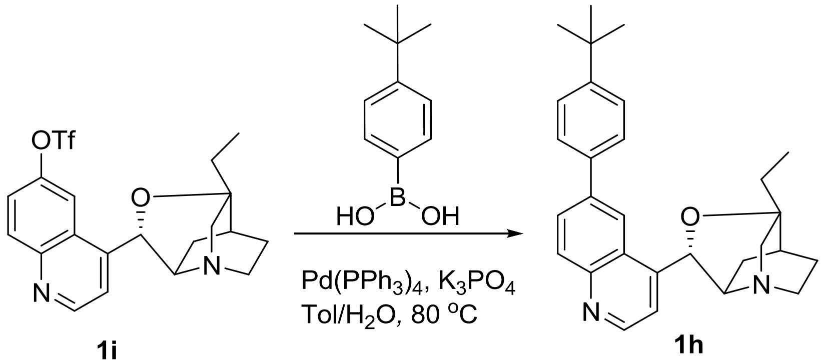

To a tube charged with $Pd(PPh_3)_4$ (21.9 mg, 0.02 mmol), K_3PO_4 (201.7 mg, 0.95 mmol,), **1i** (168.0 mg, 0.38 mmol) [1] and 4-tert-butylphenylboronic acid (101.5 mg, 0.57 mmol) was added a solution of H_2O (0.4 mL) and toluene (1.2 mL) via syringe under a nitrogen atmosphere. The mixture was heated at 80 °C until **1i** had been consumed according to TLC analysis. The mixture was cooled to room temperature, diluted with ethyl acetate, filtered, and concentrated. The crude material was purified by flash chromatography on silica gel (DCM/MeOH = 20:1) to give **1h** as

white solid (100.3 mg, 62%). $[\alpha]_D^{20}$ = +46.1 (c = 0.8 in $CHCl_3$); ^1H NMR (400 MHz, $CDCl_3$): δ = 8.92 (d, *J* = 4.8 Hz, 1H), 8.18 (d, *J* = 9.2 Hz, 1H), 8.12 (s, 1H), 7.96 (dd, *J* = 8.8 Hz, 1.6 Hz, 1H), 7.78 (d, *J* = 4.4 Hz, 1H), 7.69 (d, *J* = 8.4 Hz, 2H), 7.52 (d, *J* = 8.4 Hz, 2H), 6.12 (s, 1H), 3.64–3.58 (m, 2H), 3.04–3.02 (m, 2H), 2.71 (d, *J* = 13.6 Hz, 1H), 2.18 (t, *J* = 4.8 Hz, 1H), 1.80 (qd, *J* = 6.8 Hz, 2.0 Hz, 1H), 1.73–1.67 (m, 3H), 1.65–1.53 (m, 1H), 1.37 (s, 9H), 1.32–1.26 (m, 1H), 1.05 (t, *J* = 7.6 Hz, 3H) ppm; ^{13}C NMR (100 MHz, $CDCl_3$): δ = 150.9, 149.9, 147.2, 144.4, 139.6, 130.7, 128.9, 127.4, 126.0, 125.7, 119.9, 119.5, 110.7, 77.1, 73.0, 56.8, 54.8, 46.7, 34.6, 32.8, 31.3, 27.4, 24.0, 23.4, 7.3 ppm; ESI–HRMS: calcd. for $C_{29}H_{34}N_2O$ + H 427.2749, found 427.2749.

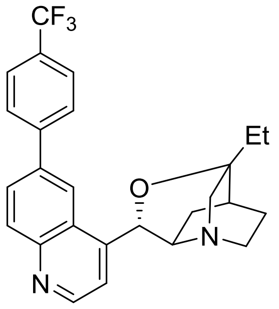

1f, 70% yield; $[\alpha]_D^{20}$ = +11.2 (*c* = 1.0 in $CHCl_3$); ^1H NMR (400 MHz, $CDCl_3$): δ = 8.94 (d, *J* = 4.4 Hz, 1H), 8.24–8.20 (m, 2H), 7.91 (dd, *J* = 8.8 Hz, 2.0 Hz, 1H), 7.80–7.78 (m, 3H), 7.68 (d, *J* = 8.0 Hz, 2H), 6.18 (s, 1H), 3.60–3.57 (m, 2H), 3.10–3.00 (m, 2H), 2.66 (d, *J* = 13.6 Hz, 1H), 2.17 (s, 1H), 1.81–1.77 (m, 1H), 1.70–1.62 (m, 3H), 1.57–1.51 (m, 1H), 1.30–1.24 (m, 1H), 1.00 (t, *J* = 7.6 Hz, 3H) ppm; ^{13}C NMR (100 MHz, $CDCl_3$): δ = 150.5, 147.6, 144.6, 143.7, 138.1, 133.9, 131.1, 128.6, 128.0, 125.8, 125.8, 125.7, 124.0, 120.9, 119.8, 77.0, 72.8, 56.9, 54.5, 46.6, 32.8, 27.3, 23.9, 23.3, 7.2 ppm; ESI–HRMS: calcd. for $C_{26}H_{25}F_3N_2O$ + H 439.1997, found 439.1996.

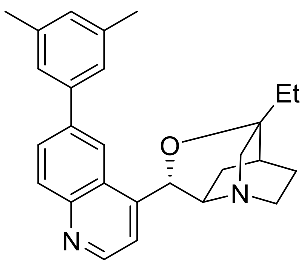

1g, 50% yield; $[\alpha]_D^{20}$ = +9.1 (*c* = 0.9 in $CHCl_3$); ^1H NMR (400 MHz, $CDCl_3$): δ = 8.93 (d, *J* = 4.4 Hz, 1H), 8.19–8.17 (m, 2H), 7.95 (dd, *J* = 8.8 Hz, 1.6 Hz, 1H), 7.78 (d, *J* = 4.0 Hz, 1H), 7.38 (s, 2H), 7.04 (s, 1H), 6.18 (s, 1H), 3.79–3.75 (m, 2H), 3.11 (s, 2H), 2.79 (d, *J* = 13.6 Hz, 1H), 2.43 (s, 6H), 2.24 (s, 1H), 1.90–1.86 (m, 1H), 1.78–1.69 (m, 3H), 1.65–1.62 (m, 1H), 1.38–1.33 (m, 1H), 1.07 (t, *J* = 7.6 Hz, 3H) ppm; ^{13}C NMR (100 MHz, $CDCl_3$): δ = 149.8, 147.3, 143.2, 140.5, 140.0, 138.5, 132.0, 130.7, 129.6, 129.5, 128.5, 125.9, 125.6, 119.9, 119.5, 77.2, 72.2, 57.1, 54.1, 46.1, 32.9, 27.3, 23.1, 22.7, 21.4, 7.3 ppm; ESI–HRMS: calcd. for $C_{27}H_{30}N_2O$ + H 399.2436, found 399.2437.

3. General procedure for assembly of MBH carbonates and *N*-silyloxycarbamates

To a solution of MBH carbonate **2a** (41.6 mg, 0.12 mmol), *N*-silyloxycarbamates **3d** (28.1 mg, 0.1

mmol) in chlorobenzene (0.5 mL) at 0 °C, catalyst **1h** (4.3 mg, 10 mol %) was added and the resulting mixture was kept at the temperature until the consumption of **3d**, as monitored by TLC analysis. Purification by flash chromatography on silica gel (AcOEt/petroleum ether = 1:15) gave **4d** as a reddish brown oil (47.0 mg, 92% yield).

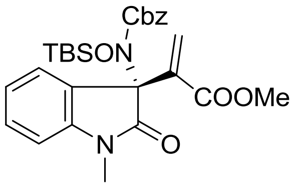

4d, 92% yield; $[\alpha]_D^{20}$ = −100.8 (*c* = 0.5 in $CHCl_3$); 91% ee, determined by HPLC analysis [Daicel Chiralpak IC, *n*-hexane/*i*-PrOH = 80/20, 1.0 mL/min, λ = 254 nm, *t* (major) = 9.463 min, *t* (minor) = 13.188 min]; ^{1}H NMR (400 MHz, $CDCl_3$): δ = 7.76（d, *J* = 6.8 Hz, 1H）, 7.32–7.27 (m, 6H), 7.01 (td, *J* = 7.6 Hz, 0.8 Hz, 1H), 6.78 (d, *J* = 7.6 Hz, 1H), 6.08 (s, 1H), 5.57 (s, 1H), 5.19 (d, *J* = 11.6 Hz, 1H), 5.11 (d, *J* = 12.0 Hz, 1H), 3.67 (s, 3H), 3.20 (s, 3H), 0.59 (s, 9H), 0.04 (s, 3H), −0.15 (s, 3H) ppm; ^{13}C NMR (100 MHz, $CDCl_3$): δ = 173.2, 165.1, 159.9, 143.8, 138.1, 135.1, 129.5, 128.8, 128.3, 128.2, 128.2, 126.9, 124.9, 122.6, 108.3, 68.5, 51.8, 26.4, 25.6, 17.9, −4.3, −4.6 ppm; ESI–HRMS: calcd. for $C_{27}H_{34}N_2O_6Si$ + Na 533.2084, found 533.2089.

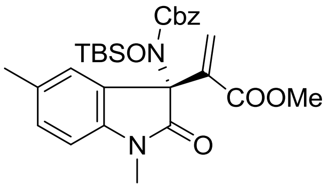

4f, 93% yield; $[\alpha]_D^{20}$ = −64.8 (*c* = 1.1 in $CHCl_3$); 91% ee, determined by HPLC analysis [Daicel Chiralpak IC, *n*-hexane/*i*-PrOH = 80/20, 1.0 mL/min, λ = 254 nm, *t* (major) = 10.204 min, *t* (minor) = 14.245 min]; ^{1}H NMR (400 MHz, $CDCl_3$): δ = 7.57 (s, 1H), 7.34–7.31 (s, 5H), 7.07 (d, *J* = 7.6 Hz, 1H), 6.67 (d, *J* = 7.6 Hz, 1H), 6.06 (s, 1H), 5.55 (s, 1H), 5.20 (d, *J* = 12.0 Hz, 1H), 5.12 (d, *J* = 12.0 Hz, 1H), 3.67 (s, 3H), 3.19 (s, 3H), 2.29 (s, 3H), 0.59 (s, 9H), 0.03 (s, 3H), −0.15 (s, 3H) ppm; ^{13}C NMR (100 MHz, $CDCl_3$): δ = 173.2, 165.1, 159.9, 141.4, 138.3, 135.1, 132.1, 129.6, 128.8, 128.3, 128.2, 128.2, 127.8, 124.6, 108.0, 68.4, 51.8, 26.4, 25.6, 21.1, 17.9, −4.3, −4.7 ppm; ESI–HRMS: calcd. for $C_{28}H_{36}N_2O_6Si$ + Na 547.2240, found 547.2239.

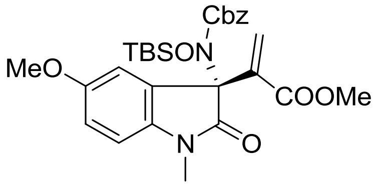

4g, 97% yield; $[\alpha]_D^{20}$ = −64.0 (*c* = 1.0 in $CHCl_3$); 94% ee, determined by HPLC analysis [Daicel Chiralpak IC, *n*-hexane/*i*-PrOH = 80/20, 1.0 mL/min, λ = 254 nm, *t* (major) = 13.032 min, *t* (minor) = 18.202 min]; ^{1}H NMR (400 MHz, $CDCl_3$): δ = 7.46 (d, *J* = 2.8 Hz, 1H), 7.31 (s, 5H), 6.81 (dd, *J* = 8.4 Hz, 2.4 Hz, 1H), 6.68 (d, *J* = 8.4 Hz, 1H), 6.06 (s, 1H), 5.56 (s, 3H), 5.19 (d, *J* = 11.6 Hz, 1H), 5.11 (d, *J* = 11.6 Hz, 1H), 3.74 (s, 3H),

3.66 (s, 3H), 3.17 (s, 3H), 0.60 (s, 9H), 0.05 (s, 3H), −0.10 (s, 3H) ppm; ^{13}C NMR (100 MHz, $CDCl_3$): δ = 173.0, 165.0, 159.8, 155.8, 138.1, 137.2, 135.1, 129.4, 128.8, 128.2, 128.2, 124.9, 114.2, 114.0, 108.6, 75.6, 68.4, 55.7, 51.8, 26.4, 25.6, 17.9, −4.2, −4.5 ppm; ESI–HRMS: calcd. for $C_{28}H_{36}N_2O_7Si$ + Na 563.2189, found 563.2187.

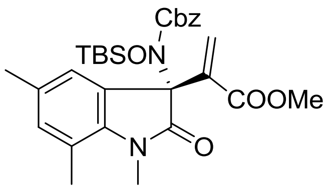

4h, 93% yield; $[\alpha]_D^{20}$ = −60.0 (*c* = 0.8 in $CHCl_3$); 90% ee, determined by HPLC analysis [Daicel Chiralpak IC, *n*-hexane/*i*-PrOH = 80/20, 1.0 mL/min, λ = 254 nm, *t* (major) = 12.702 min, *t* (minor) = 15.971 min]; ^{1}H NMR (400 MHz, $CDCl_3$): δ = 7.41 (s, 1H), 7.33 (s, 5H), 6.81 (s, 1H), 6.06 (s, 1H), 5.54 (s, 1H), 5.18 (d, *J* = 12.0 Hz, 1H), 5.12 (d, *J* = 11.6 Hz, 1H), 3.66 (s, 3H), 3.44 (s, 3H), 2.46 (s, 3H), 2.23 (s, 3H), 0.62 (s, 9H), 0.04 (s, 3H), −0.14 (s, 3H) ppm; ^{13}C NMR (100 MHz, $CDCl_3$): δ = 174.1, 165.1, 160.0, 139.1, 138.5, 135.2, 133.6, 131.9, 129.1, 128.8, 128.2, 128.1, 125.5, 124.8, 119.4, 74.5, 68.4, 51.8, 29.9, 25.6, 20.8, 18.9, 17.9, −4.2, −4.7 ppm; ESI–HRMS: calcd. for $C_{29}H_{38}N_2O_6Si$ + Na 561.2397, found 561.2394.

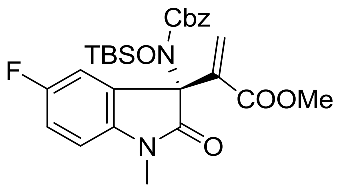

4i, 86% yield; $[\alpha]_D^{20}$ = −64.3 (*c* = 1.1 in $CHCl_3$); 90% ee, determined by HPLC analysis [Daicel Chiralpak IC, *n*-hexane/*i*-PrOH = 80/20, 1.0 mL/min, λ = 254 nm, *t* (major) = 7.288 min, *t* (minor) = 8.890 min]; ^{1}H NMR (400 MHz, $CDCl_3$): δ = 7.58 (dd, *J* = 8.4 Hz, 2.4 Hz, 1H), 7.32 (s, 5H), 6.99 (td, *J* = 8.4 Hz, 2.4 Hz, 1H), 6.70 (dd, *J* = 8.4 Hz, 4.0 Hz, 1H), 6.10 (s, 1H), 5.60 (s, 1H), 5.19 (d, *J* = 12.0 Hz, 1H), 5.11 (d, *J* = 12.0 Hz, 1H), 3.68 (s, 3H), 3.20 (s, 3H), 0.61 (s, 9H), 0.04 (s, 3H), -0.10 (s, 3H) ppm; ^{13}C NMR (100 MHz, $CDCl_3$): δ = 173.1, 164.9, 160.0 (d, $^{1}J_{C,F}$ = 239.1 Hz), 159.6, 139.8, 137.6, 134.9, 129.8, 128.9, 128.3, 128.3, 125.4, 115.6 (d, $^{2}J_{C,F}$ = 23.5 Hz), 115.4 (d, $^{2}J_{C,F}$ = 26.1 Hz), 108.6 (d, $^{3}J_{C,F}$ = 8.0 Hz), 68.6, 52.0, 26.6, 25.6, 17.9, -4.3, -4.6 ppm; ESI–HRMS: calcd. for $C_{27}H_{33}FN_2O_6Si$ + Na 551.1990, found 551.1993.

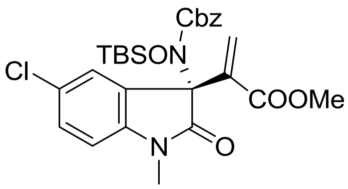

4j, 88% yield; $[\alpha]_D^{20}$ = −62.8 (*c* = 1.1 in $CHCl_3$); 89% ee, determined by HPLC analysis [Daicel Chiralpak IC, *n*-hexane/*i*-PrOH = 80/20, 1.0 mL/min, λ = 254 nm, *t* (major) = 7.504 min, *t* (minor) = 9.269 min]; ^{1}H NMR (400 MHz, $CDCl_3$): δ = 7.79 (d, *J* = 2.0 Hz, 1H), 7.32 (s, 5H), 7.26 (dd, *J* = 8.4 Hz, 2.4 Hz, 1H), 6.70 (d, *J* = 8.4 Hz, 1H), 6.10 (s, 1H), 5.59 (s, 1H), 5.19

(d, J = 11.6 Hz, 1H), 5.11 (d, J = 11.6 Hz, 1H), 3.67 (s, 3H), 3.20 (s, 3H), 0.61 (s, 9H), 0.04 (s, 3H), −0.11 (s, 3H) ppm; ^{13}C NMR (100 MHz, $CDCl_3$): δ = 173.0, 164.8, 159.6, 142.4, 137.6, 134.9, 129.9, 129.3, 128.9, 128.3, 128.3, 128.0, 127.5, 125.3, 109.1, 68.6, 52.0, 26.5, 25.6, 17.9, −4.3, −4.6 ppm; ESI–HRMS: calcd. for $C_{27}H_{33}ClN_2O_6Si$ + Na 567.1694, found 567.1698.

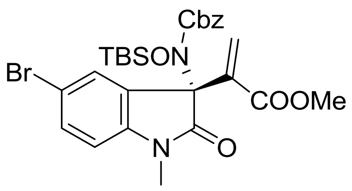

4k, 81% yield; $[\alpha]_D^{20}$ = −54.6 (c = 0.9 in $CHCl_3$); 88% ee, determined by HPLC analysis [Daicel Chiralpak IC, n-hexane/i-PrOH = 80/20, 1.0 mL/min, λ = 254 nm, t (major) = 7.186 min, t (minor) = 8.624 min]; ^{1}H NMR (400 MHz, $CDCl_3$): δ = 7.91 (d, J = 2.0 Hz, 1H), 7.40 (dd, J = 8.0 Hz, 2.0 Hz, 1H), 7.32 (s, 5H), 6.65 (d, J = 8.4 Hz, 1H), 6.10 (s, 1H), 5.59 (s, 1H), 5.19 (d, J = 11.6 Hz, 1H), 5.10 (d, J = 11.6 Hz, 1H), 3.67 (s, 3H), 3.19 (s, 3H), 0.59 (s, 9H), 0.03 (s, 3H), −0.11 (s, 3H) ppm; ^{13}C NMR (100 MHz, $CDCl_3$): δ = 172.9, 164.8, 159.6, 142.9, 137.6, 134.9, 132.2, 130.2, 128.9, 128.3, 128.3, 125.3, 115.4, 109.7, 74.8, 68.6, 52.0, 26.5, 25.6, 17.9, −4.3, −4.6 ppm; ESI–HRMS: calcd. for $C_{27}H_{33}BrN_2O_6Si$ + Na 611.1189, found 611.1195.

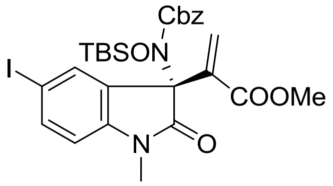

4l, 85% yield; $[\alpha]_D^{20}$ = −57.5 (c = 1.2 in $CHCl_3$); 86% ee, determined by HPLC analysis [Daicel Chiralpak IC, n-hexane/i-PrOH = 80/20, 1.0 mL/min, λ = 254 nm, t (major) = 7.946 min, t (minor) = 9.788 min]; ^{1}H NMR (400 MHz, $CDCl_3$): δ = 8.07 (d, J = 1.6 Hz, 1H), 7.60 (dd, J = 8.4 Hz, 2.0 Hz, 1H), 7.32 (s, 5H), 6.56 (d, J = 8.4 Hz, 1H), 6.10 (s, 1H), 5.58 (s, 1H), 5.19 (d, J = 12.0 Hz, 1H), 5.11 (d, J = 11.6 Hz, 1H), 3.67 (s, 3H), 3.20 (s, 3H), 0.60 (s, 9H), 0.03 (s, 3H), −0.10 (s, 3H) ppm; ^{13}C NMR (100 MHz, $CDCl_3$): δ = 172.7, 164.8, 159.6, 143.6, 138.2, 137.6, 135.6, 134.9, 130.6, 128.9, 128.3, 128.3, 125.3, 110.3, 68.6, 52.0, 26.5, 25.6, 17.9, −4.3, −4.6 ppm; ESI–HRMS: calcd. for $C_{27}H_{33}IN_2O_6Si$ + Na 659.1050, found 659.1046.

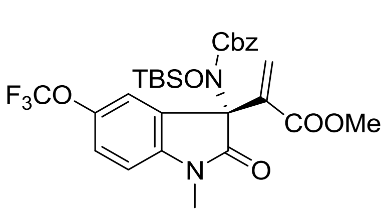

4m, 71% yield; $[\alpha]_D^{20}$ = −59.4 (c = 0.6 in $CHCl_3$); 85% ee, determined by HPLC analysis [Daicel Chiralpak IC, n-hexane/i-PrOH = 80/20, 1.0 mL/min, λ = 254 nm, t (major) = 5.957 min, t (minor) = 6.527 min]; ^{1}H NMR (400 MHz, $CDCl_3$): δ = 7.74 (s, 1H), 7.31 (s, 5H), 7.16 (d, J = 8.4 Hz, 1H), 6.75 (d, J = 8.4 Hz, 1H), 6.11 (s, 1H), 5.60 (s,

1H), 5.18 (d, J = 11.6 Hz, 1H), 5.09 (d, J = 11.6 Hz, 1H), 3.67 (s, 3H), 3.21 (s, 3H), 0.60 (s, 9H), 0.04 (s, 3H), −0.13 (s, 3H) ppm; ^{13}C NMR (100 MHz, $CDCl_3$): δ = 173.1, 164.9, 159.5, 144.6, 142.5, 137.5, 134.9, 129.7, 128.9, 128.3, 128.3, 125.6, 122.5, 121.1, 119.2, 108.6, 75.0, 68.7, 52.0, 26.6, 25.6, 17.9, −4.2, −4.9 ppm; ESI–HRMS: calcd. for $C_{28}H_{33}F_3N_2O_7Si$ + Na 617.1907, found 617.1912.

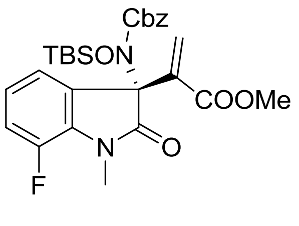

4n, 85% yield; $[\alpha]_D^{20}$ = −49.5 (c = 0.5 in $CHCl_3$); 90% ee, determined by HPLC analysis [Daicel Chiralpak IC, n-hexane/i-PrOH = 80/20, 1.0 mL/min, λ = 254 nm, t (major) = 6.892 min, t (minor) = 8.365 min]; ^{1}H NMR (400 MHz, $CDCl_3$): δ = 7.55 (dd, J = 7.6 Hz, 0.8 Hz, 1H), 7.34-7.30 (m, 5H), 7.04–7.00 (m, 1H), 6.95–6.90 (m, 1H), 6.12 (s, 1H), 5.61 (s, 1H), 5.17 (d, J = 12.0 Hz, 1H), 5.11 (d, J = 12.0 Hz, 1H), 3.66 (s, 3H), 3.40 (d, J = 2.8 Hz, 3H), 0.64 (s, 9H), 0.05 (s, 3H), −0.13 (s, 3H) ppm; ^{13}C NMR (100 MHz, $CDCl_3$): δ = 173.0, 165.0, 159.6, 147.8 (d, $^1J_{C,F}$ = 241.7 Hz), 137.8, 135.0, 131.1, 130.6, 128.9, 128.3, 128.3, 125.7, 123.0 (d, $^3J_{C,F}$ = 6.3 Hz), 122.7 (d, $^4J_{C,F}$ = 3.5 Hz), 117.5 (d, $^2J_{C,F}$ = 19.0 Hz), 77.2, 68.6, 51.9, 29.0 (d, $^3J_{C,F}$ = 6.3 Hz), 25.7, 18.0, −4.2, −4.5 ppm; ESI–HRMS: calcd. for $C_{27}H_{33}FN_2O_6Si$ + Na 551.1990, found 551.1996.

4. Synthetic transformations of multifunctional adduct 4d

Zinc powder (84.5 mg, 1.3 mmol) was added to a stirred solution of **4d** (25.5 mg, 0.05 mmol) in AcOH/H_2O/THF (3:1:1) (0.5 mL) under a nitrogen atmosphere. The mixture was stirred at room temperature for 0.5h, and then stirred at 60 °C until the consumption of **4d**. The mixture was diluted with water and filtered. The filtrate was extracted with Et_2O (3 × 5 mL), and the aqueous layer was washed with aq NaOH (6.0 M) until pH 7, and the aqueous layer was extracted with Et_2O (3 × 5 mL). The combined organic layers were dried with anhydrous Na_2SO_4 and concentrated. Flash chromatography on silica gel (petroleum ether/ethyl acetate = 4:1) afforded pure product **5** as a white solid (6.7 mg, 35%). $[\alpha]_D^{20}$ = −64.7 (c = 0.9 in $CHCl_3$); 90% ee, determined by HPLC analysis [Daicel Chiralcel OD, n-hexane/i-PrOH = 80/20, 1.0 mL/min, λ = 254 nm, t (minor) = 13.183 min, t (major) = 19.307 min]; ^{1}H NMR (400 MHz, $CDCl_3$): δ = 7.44 (d, J = 7.6 Hz, 1H), 7.34–7.29 (m, 4H), 7.25–7.22 (m, 2H), 7.05 (t, J = 7.6 Hz, 1H), 6.83 (d, J = 8.4 Hz, 1H), 6.57 (s, 1H), 6.32 (s, 1H), 5.87 (s, 1H), 4.97 (s, 2H), 3.73 (s, 3H), 3.23 (s, 3H) ppm; ^{13}C NMR (100 MHz, $CDCl_3$): δ = 174.2, 166.0, 154.6, 143.8, 136.3, 135.7, 129.6, 128.7, 128.4, 128.1, 128.1, 128.1,

124.6, 122.9 108.5, 67.2, 64.1, 52.5, 26.7 ppm; ESI–HRMS: calcd. for $C_{21}H_{20}N_2O_5$ + Na 403.1270, found 403.1271.

HF·pyridine (5.4 μL, 0.06 mmol) was added to a solution of **4d** (25.5 mg, 0.05 mmol) in THF (0.3 mL) at 0 °C. The mixture was stirred at room temperature until the consumption of **4d**. Then saturated $NaHCO_3$ was added until pH 7, and the aqueous layer was extracted with Et_2O (3 × 3.0 mL). The combined organic layers were dried with anhydrous Na_2SO_4 and concentrated. Flash chromatography on silica gel (petroleum ether/ethyl acetate = 3:1) afforded pure product **6** as a colorless oil (17.8 mg, 90%). K_2CO_3 (1.4 mg, 0.01 mmol) was added to a solution of **6** in THF (0.3 mL) at 0 °C. The mixture was stirred at room temperature until **6** was consumed. Then the reaction mixture was concentrated in vacuo at low temperature and purified by flash chromatography on silica gel (petroleum ether/ethyl acetate = 5:1) afforded pure product **7** as a white semisolid (9.5 mg, 53%). $[\alpha]_D^{20}$ = +8.8 (*c* = 0.5 in $CHCl_3$); 90% ee, determined by HPLC analysis [Daicel Chiralcel OD, *n*-hexane/*i*-PrOH = 80/20, 1.0 mL/min, λ = 254 nm, *t* (minor) = 16.310 min, *t* (major) = 19.092 min]; ^{1}H NMR (400 MHz, $CDCl_3$): δ = 7.41 (t, *J* = 7.2 Hz, 1H), 7.39–7.26 (m, 4H), 7.17–7.13 (m, 3H), 6.82 (d, *J* = 7.6 Hz, 1H), 6.43 (s, 1H), 5.40 (s, 1H), 5.18 (d, *J* = 12.0 Hz, 1H), 5.07 (d, *J* = 12.0 Hz, 1H), 3.02 (s, 3H) ppm; ^{13}C NMR (100 MHz, $CDCl_3$): δ = 172.0, 162.6, 153.6, 143.8, 135.0, 134.1, 130.9, 128.6, 128.5, 128.2, 126.7, 126.2, 124.1, 124.0, 109.1, 72.0, 69.1, 26.5 ppm; ESI–HRMS: calcd. for $C_{20}H_{16}N_2O_5$ + Na 387.0957, found 387.0959.

5. Crystal data and structure refinement for enantiopure 5

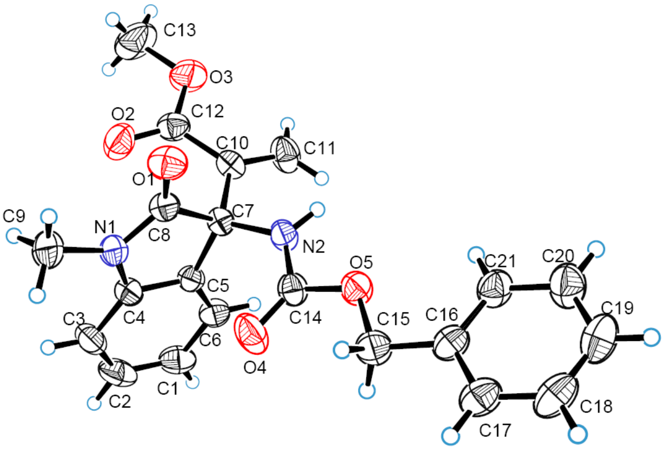

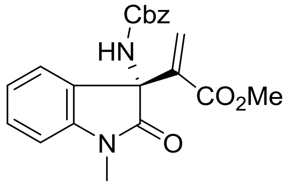

5

Identification code	5
Empirical formula	$C_{21}H_{20}O_5N_2$
Formula weight	380.39
Temperature	291(2)
Crystal system	Orthorhombic
Space group	$P2_12_12_1$
a/Å, b/Å, c/Å	8.6032(2), 9.40760(10), 23.8035(4)
α/°, β/°, γ/°,	90.00, 90.00, 90.00
Volume/Å^3	1926.55(6)

Z	4
ρ_{calc}mg/mm^3	1.311
m/mm^{-1}	0.782
F(000)	800
Crystal size	0.42 × 0.36 × 0.30
Theta range for data collection	5.06 to 69.79°
Index ranges	$-10 \leq h \leq 10$, $-11 \leq k \leq 11$, $-28 \leq l \leq 28$
Reflections collected	15142
Independent reflections	3601[R(int) = 0.0224]
Data/restraints/parameters	3601/0/256
Goodness-of-fit on F^2	1.041
Final R indexes [I>2σ (I)]	$R_1 = 0.0279$, $wR_2 = 0.0782$
Final R indexes [all data]	$R_1 = 0.0289$, $wR_2 = 0.0793$

Largest diff. peak/hole 0.141/-0.109

6. NMR spectra and HPLC chromatograms

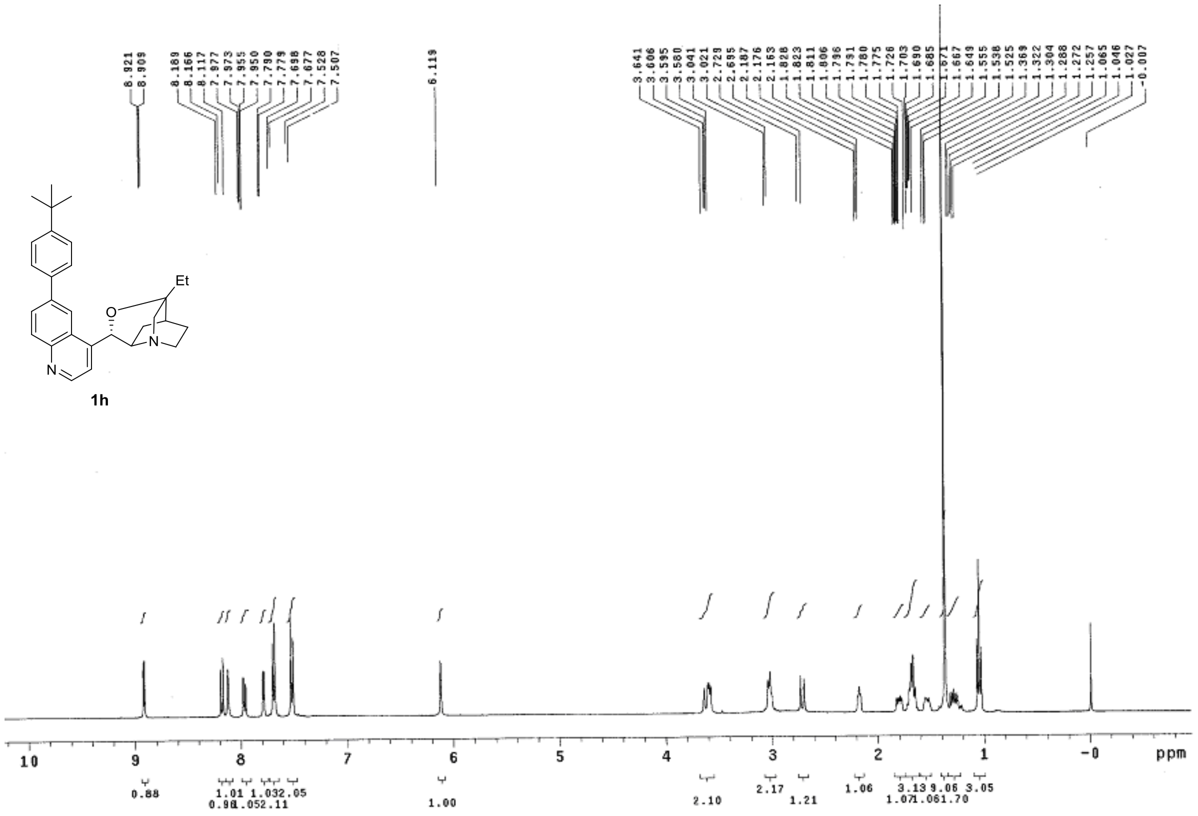
1h

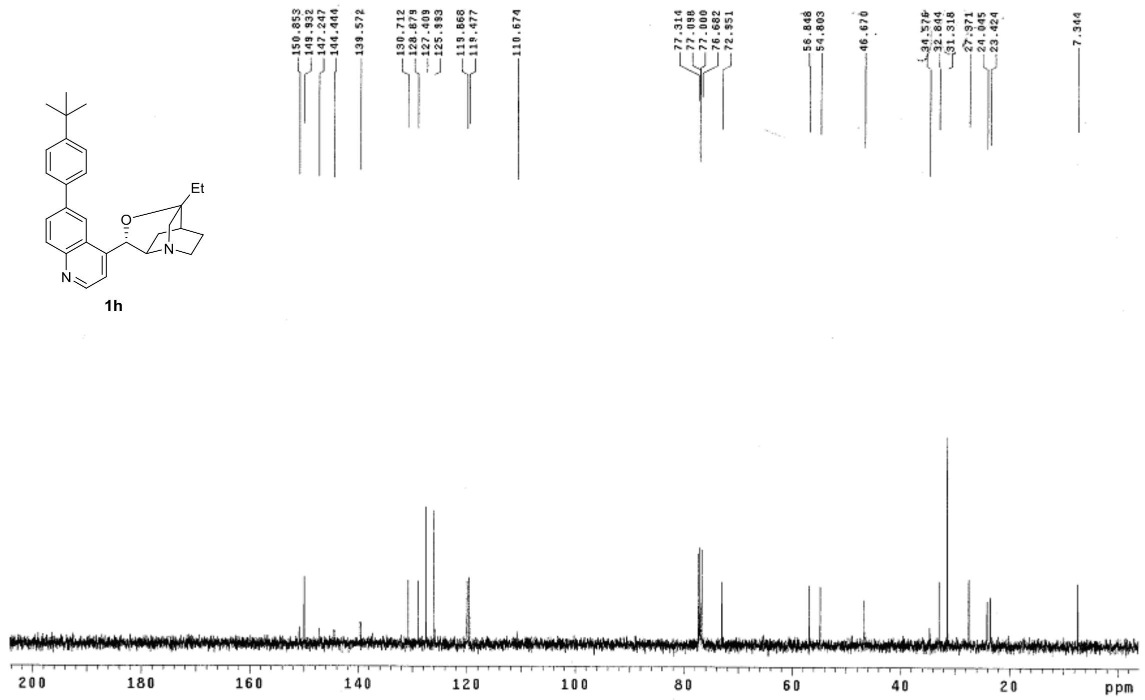

1h

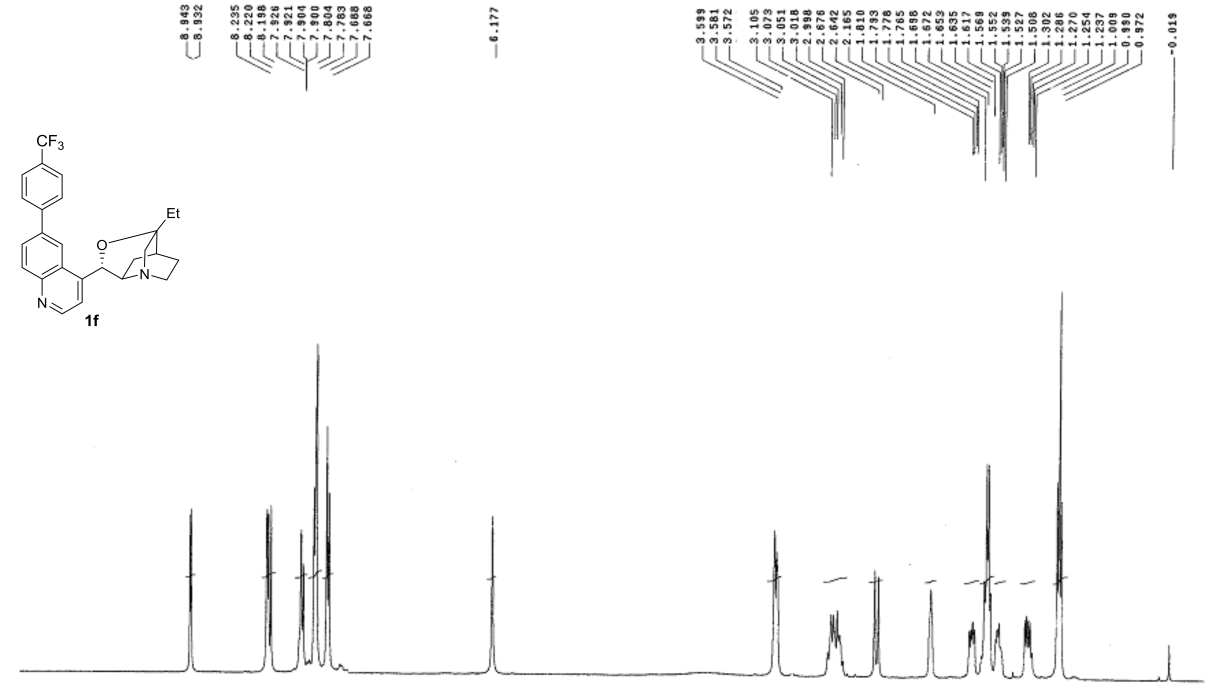

1f

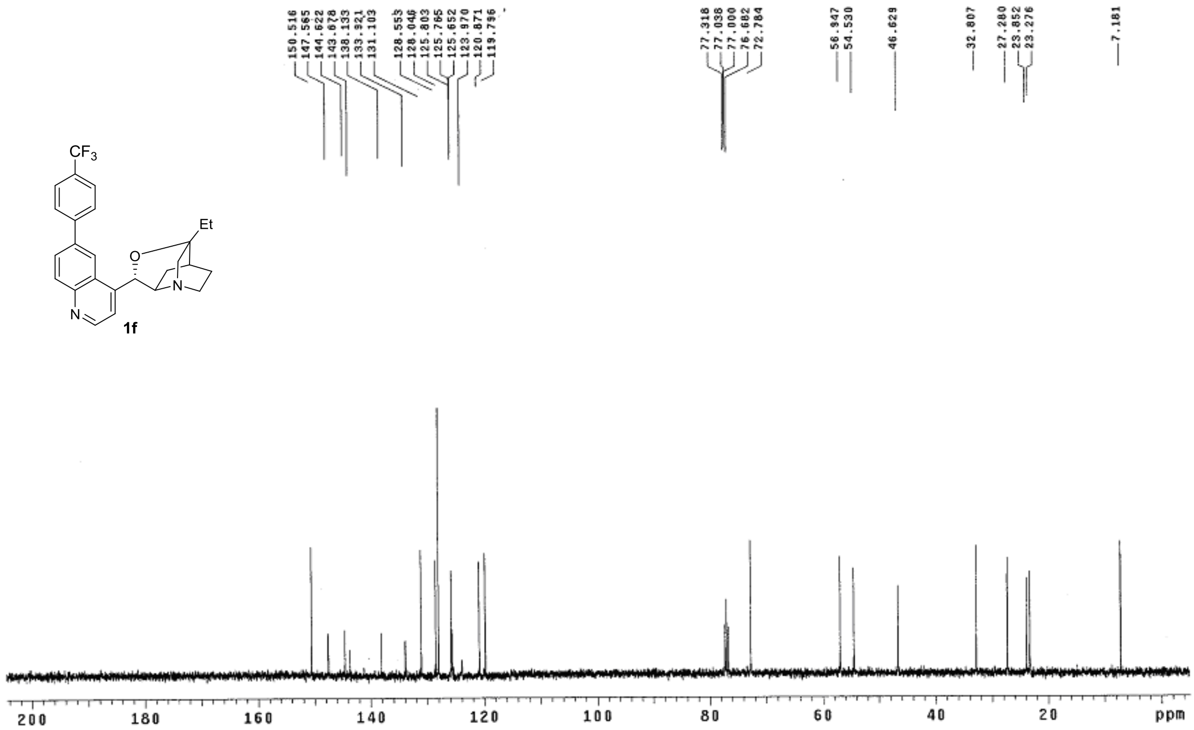
CF3
Et
O
N
1f
ppm

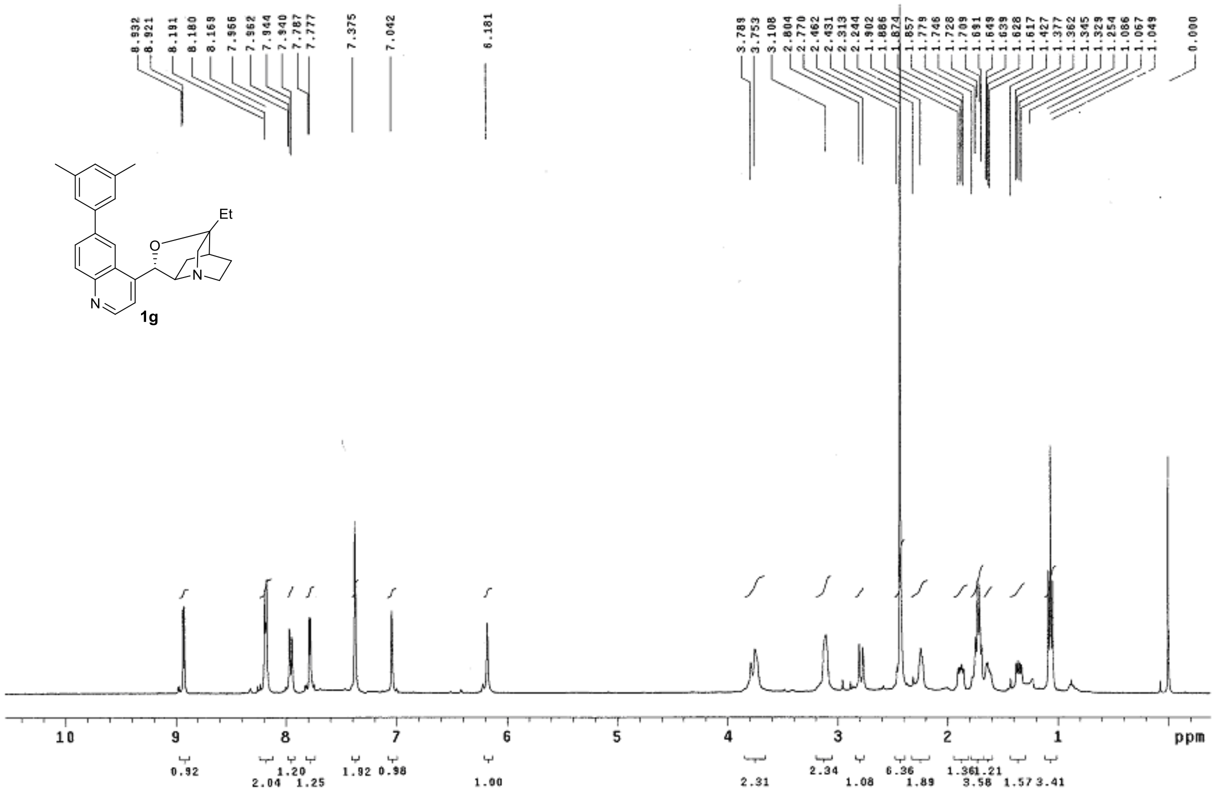
Et
O
N
1g
ppm

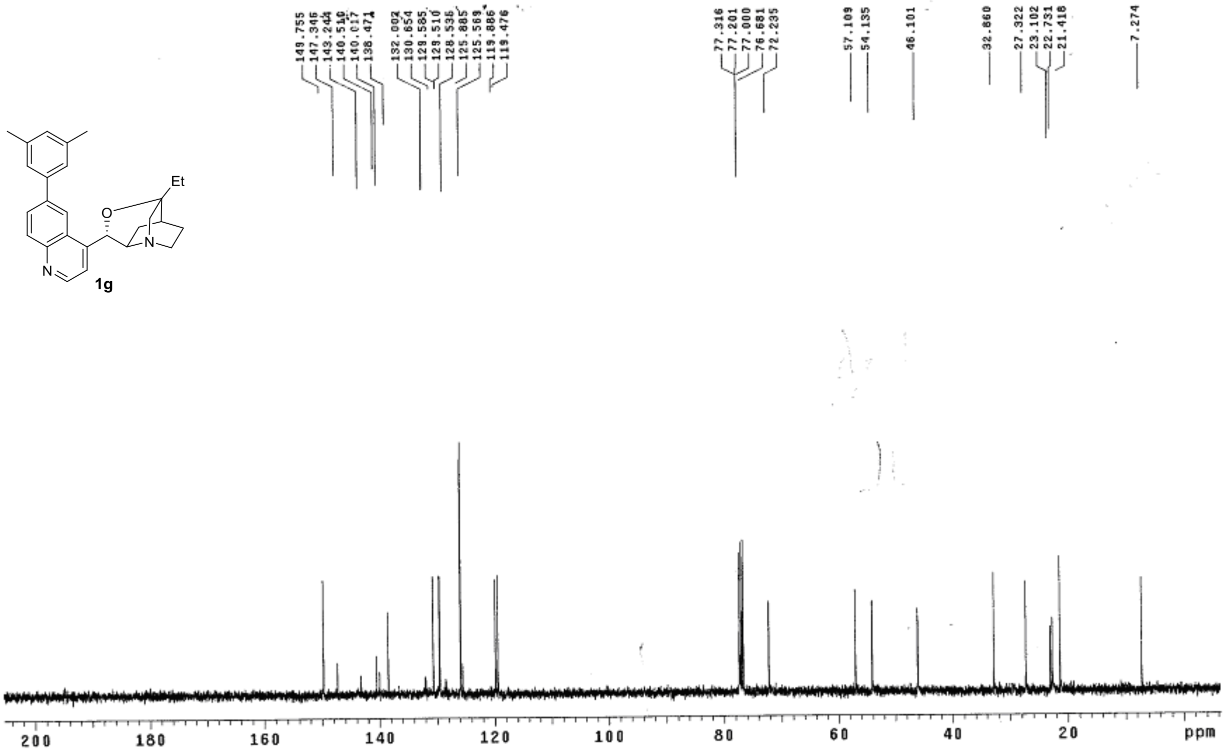

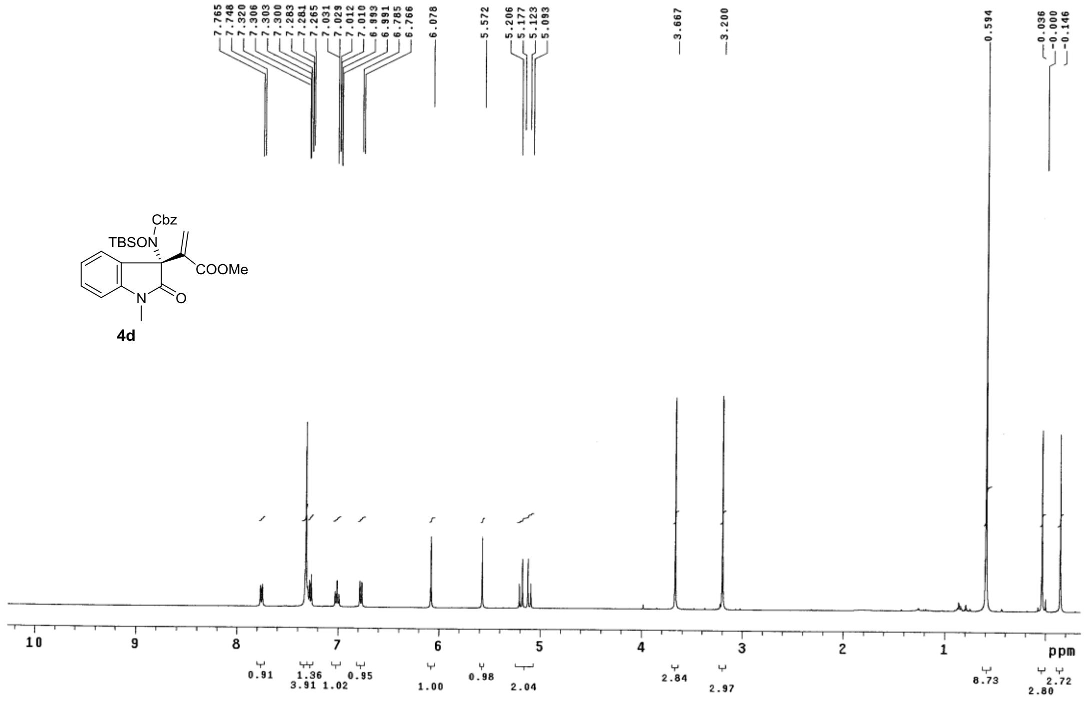
TBSON
Cbz
COOMe
N
O
4d
ppm

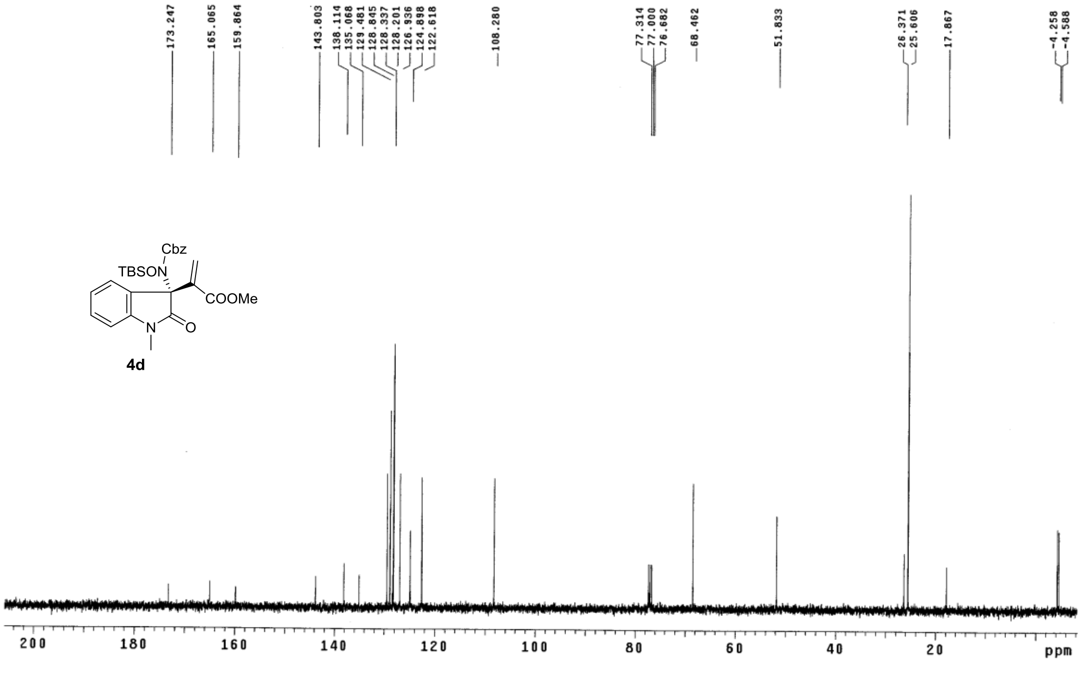
TBSON
Cbz
COOMe
N
O
4d
ppm

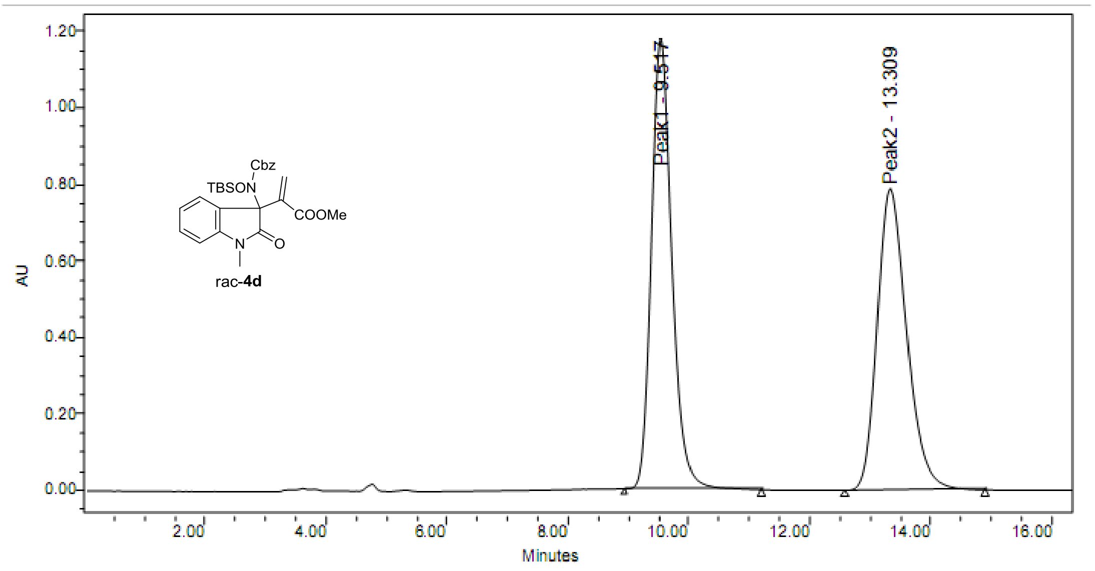

	Peak Name	RT (min)	Area (µV*sec)	% Area	Height (µV)	% Height
1	Peak1	9.517	27486208	50.61	1177591	59.94
2	Peak2	13.309	26821039	49.39	787107	40.06

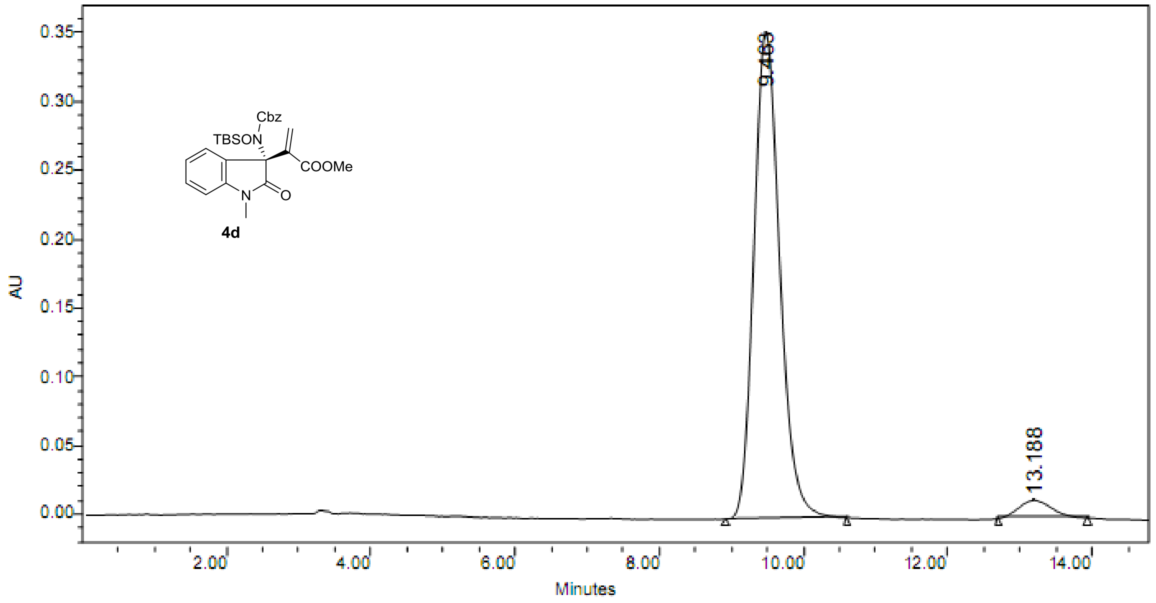

	RT (min)	Area (µV*sec)	% Area	Height (µV)	% Height
1	9.463	8818105	95.54	353863	96.46
2	13.188	411939	4.46	12985	3.54

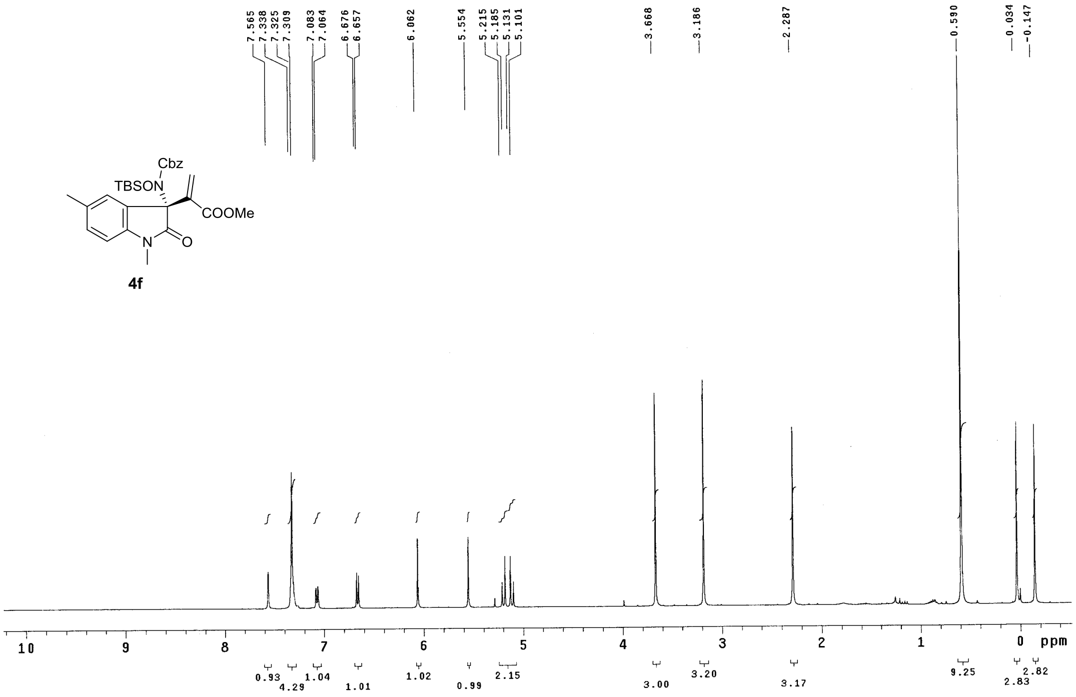
7.565
7.338
7.325
7.309
7.083
7.064
6.676
6.657
6.062
5.554
5.215
5.185
5.131
5.101
3.668
3.186
2.287
0.590
0.034
-0.147
Cbz
TBSON
COOMe
N
O
4f
10 9 8 7 6 5 4 3 2 1 0 ppm
0.93
4.29
1.04
1.01
1.02
0.99
2.15
3.00
3.20
3.17
9.25
2.83
2.82

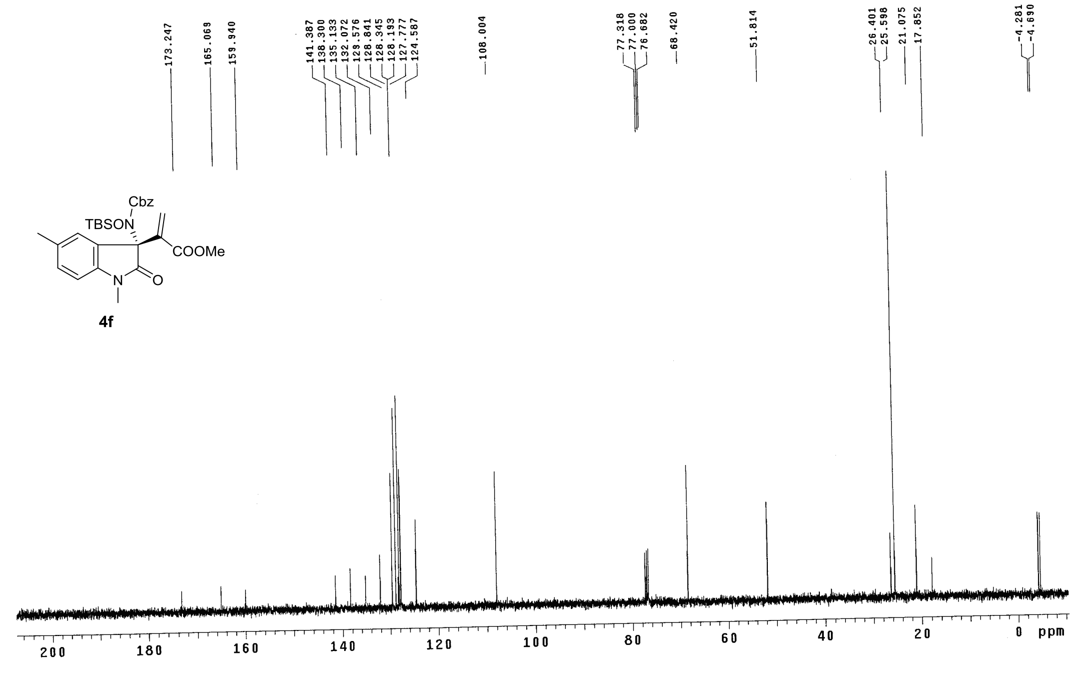
173.247
165.069
159.940
141.387
138.300
135.133
132.072
129.576
128.841
128.345
128.193
127.777
124.587
108.004
77.318
77.000
76.682
68.420
51.814
26.401
25.598
21.075
17.852
-4.281
-4.690
Cbz
TBSON
COOMe
N
O
4f
200 180 160 140 120 100 80 60 40 20 0 ppm

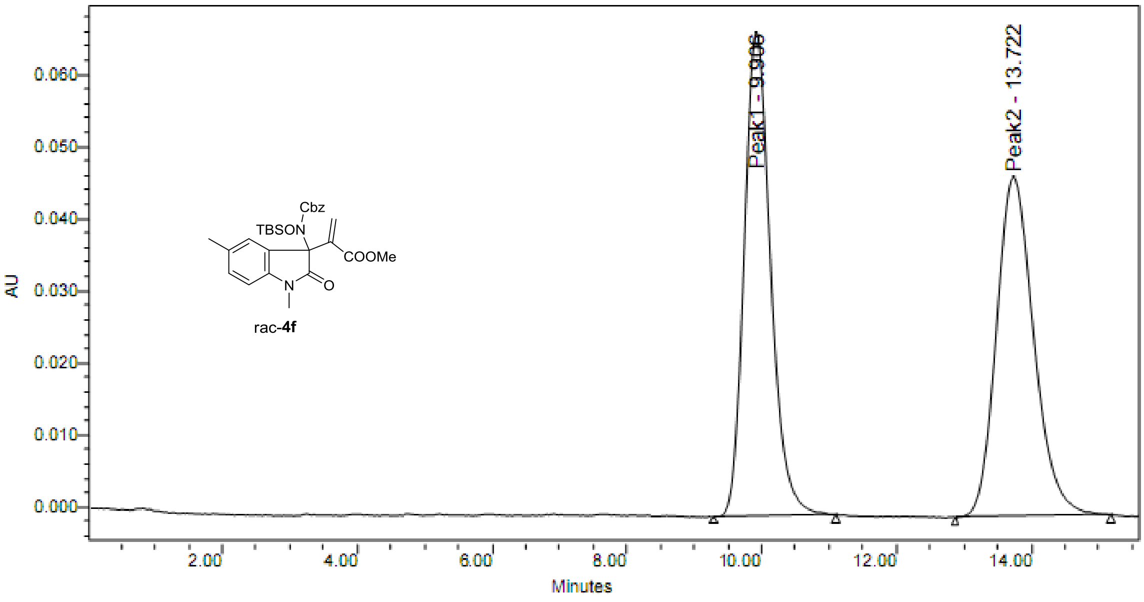

	Peak Name	RT (min)	Area (µV*sec)	% Area	Height (µV)	% Height
1	Peak1	9.906	1830681	50.07	67226	58.79
2	Peak2	13.722	1825506	49.93	47116	41.21

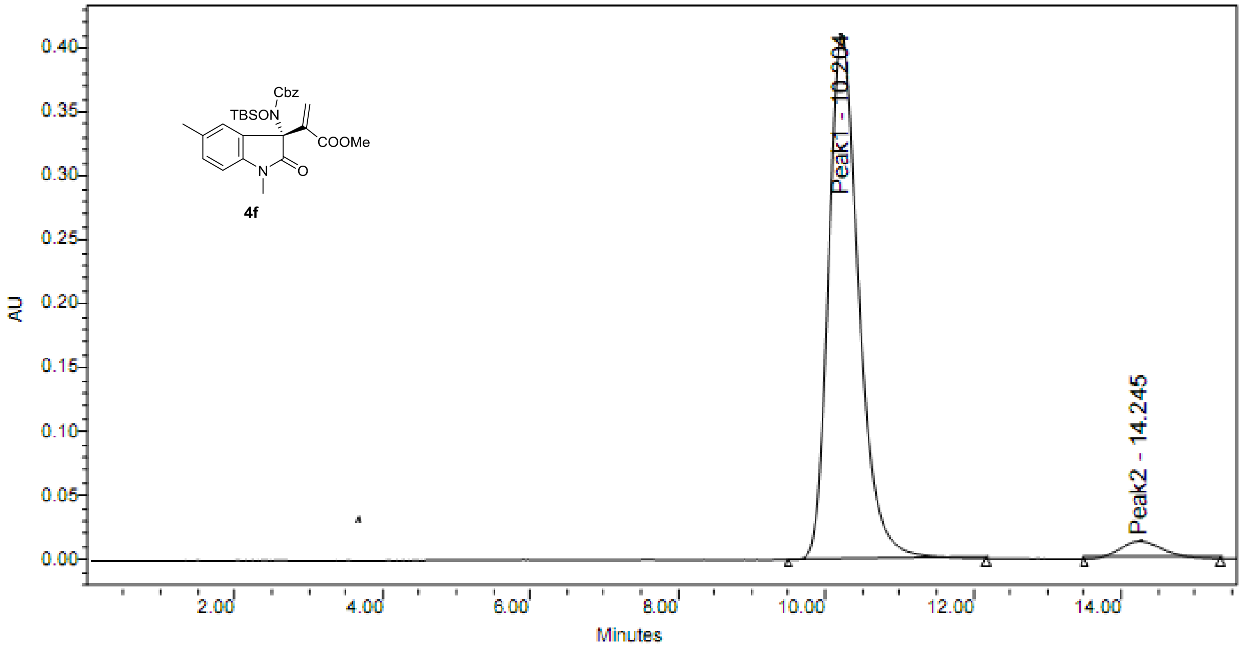

	Peak Name	RT (min)	Area (µV*sec)	% Area	Height (µV)	% Height
1	Peak1	10.204	11722999	95.43	411002	96.80
2	Peak2	14.245	561774	4.57	13595	3.20

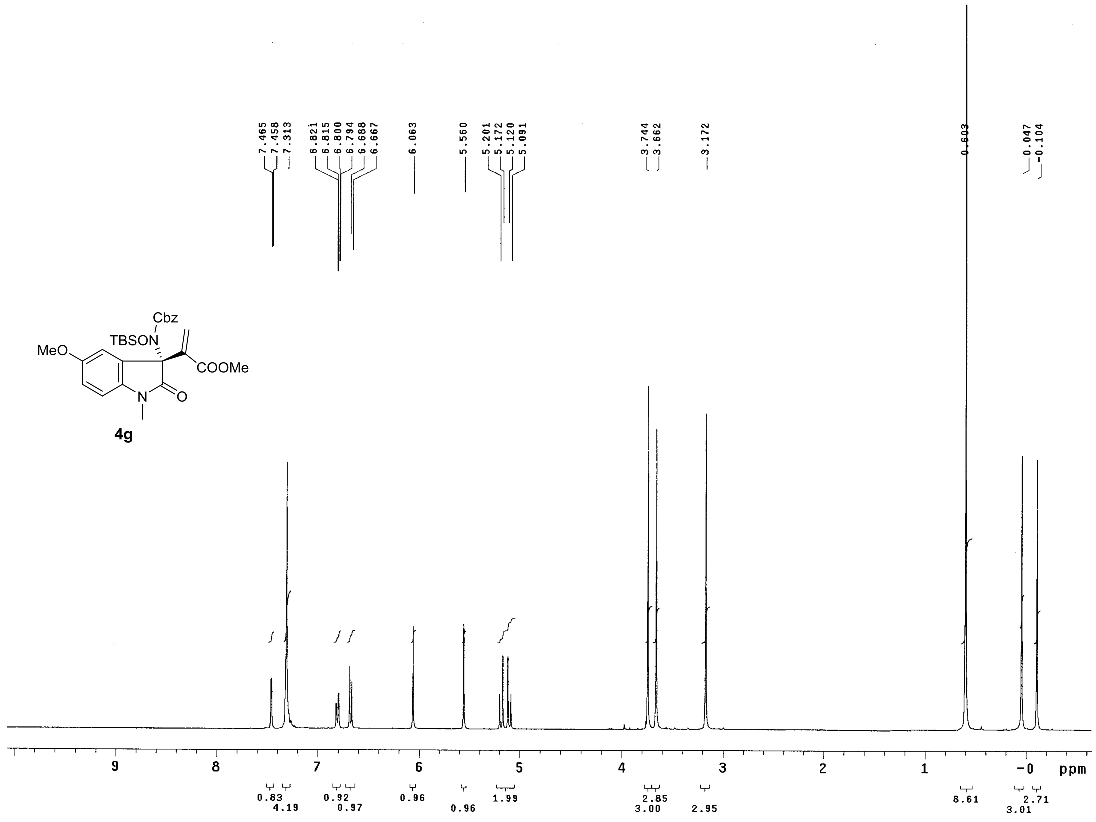

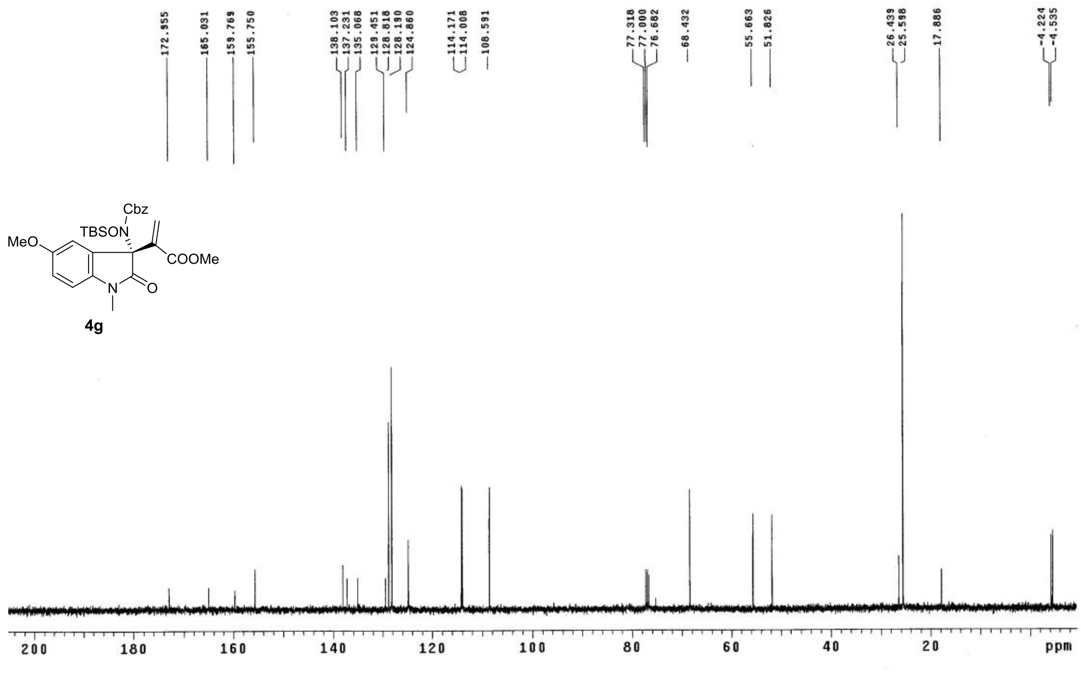

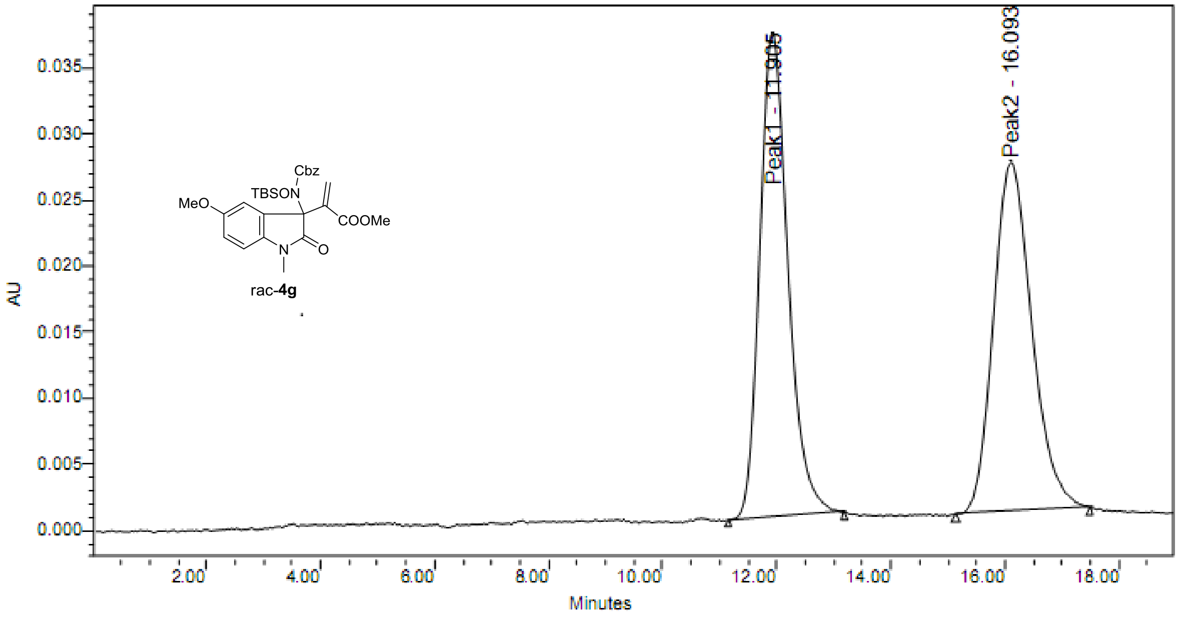

	Peak Name	RT (min)	Area (µV*sec)	% Area	Height (µV)	% Height
1	Peak1	11.905	1263055	50.37	36689	58.10
2	Peak2	16.093	1244462	49.63	26454	41.90

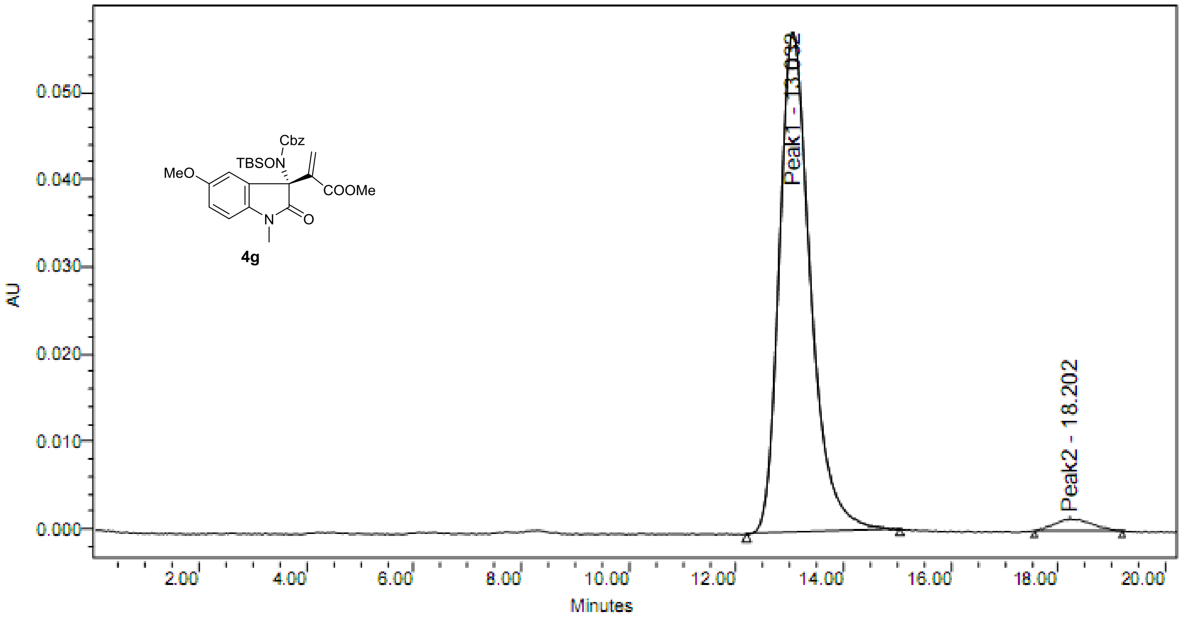

	Peak Name	RT (min)	Area (µV*sec)	% Area	Height (µV)	% Height
1	Peak1	13.032	2326947	97.15	57280	97.68
2	Peak2	18.202	68295	2.85	1362	2.32

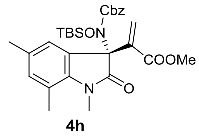
Cbz
TBSON
COOMe
N
O

4h

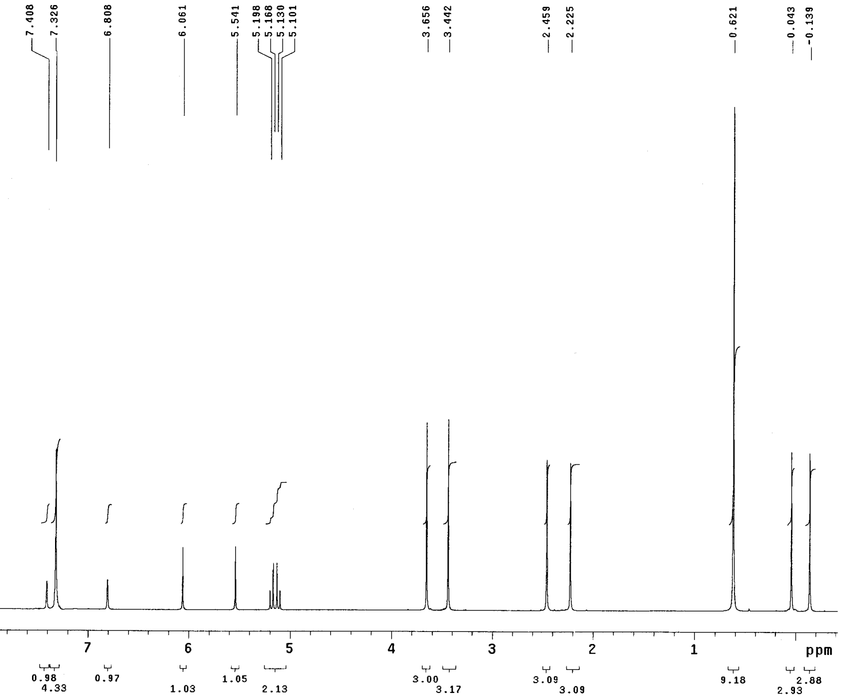
7.408
7.326
6.808
6.061
5.541
5.198
5.168
5.130
5.101
3.656
3.442
2.459
2.225
0.621
0.043
-0.139
ppm
0.98
4.33
0.97
1.03
1.05
2.13
3.00
3.17
3.09
3.09
9.18
2.93
2.88

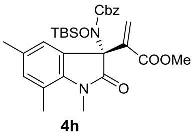
Cbz
TBSON
COOMe
N
O

4h

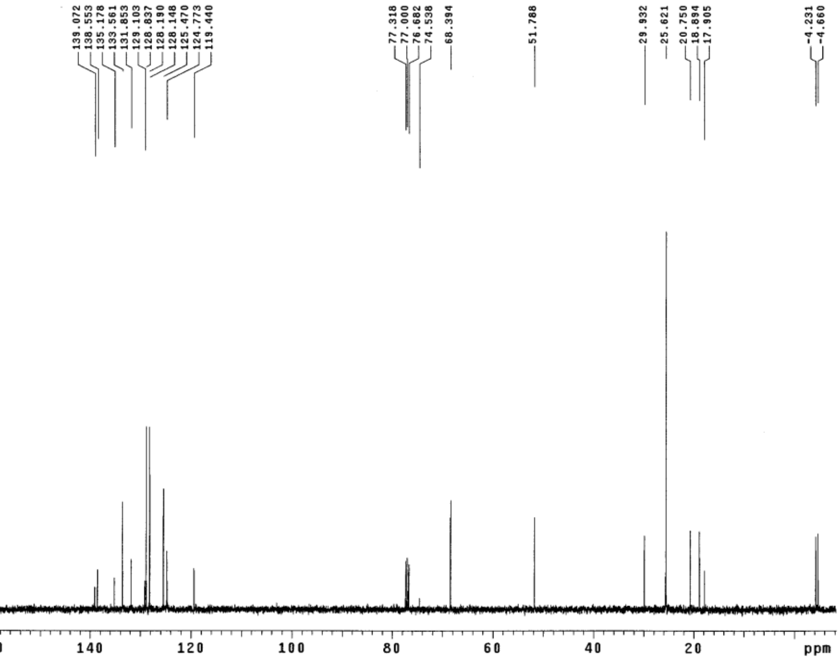
174.141
165.099
159.970
139.072
138.553
135.178
133.561
131.853
129.103
128.837
128.190
128.148
125.470
124.773
119.440
77.318
77.000
76.682
74.538
68.394
51.788
29.932
25.621
20.750
18.894
17.905
-4.231
-4.660
ppm

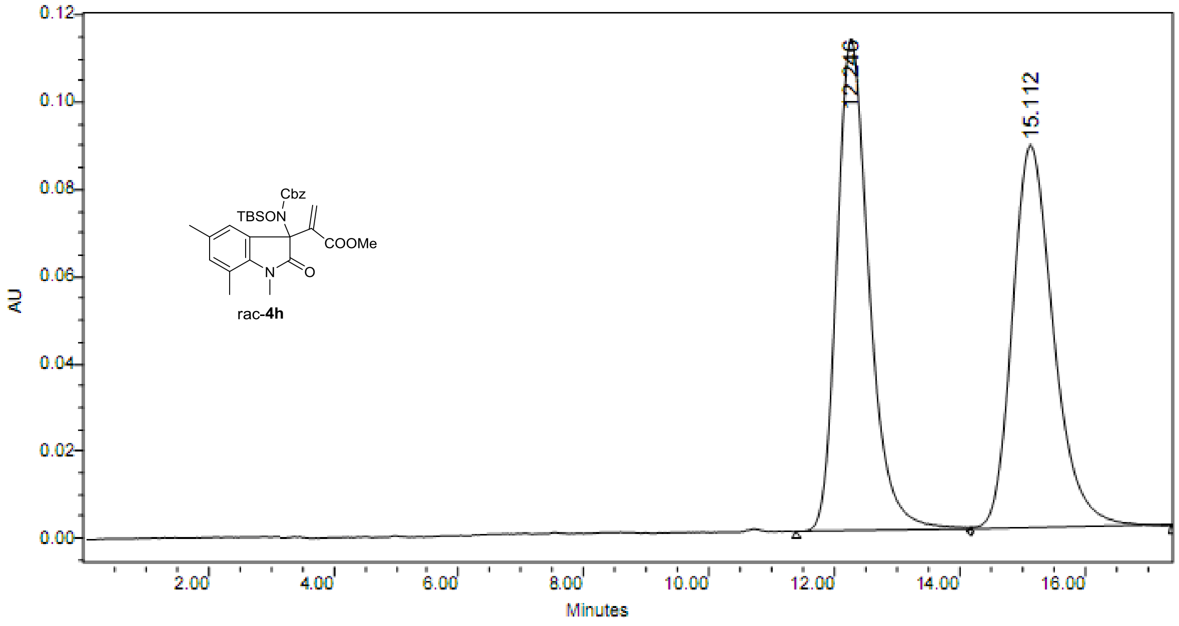

	RT (min)	Area (µV*sec)	% Area	Height (µV)	% Height
1	12.246	4049174	50.11	112615	56.24
2	15.112	4032010	49.89	87638	43.76

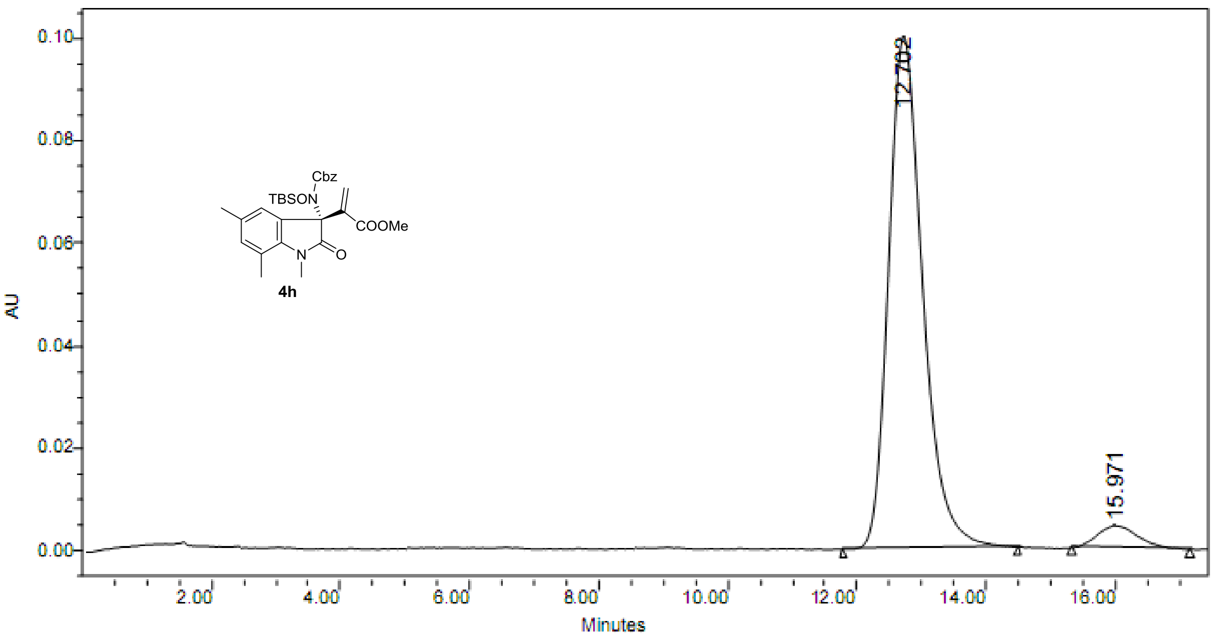

	RT (min)	Area (µV*sec)	% Area	Height (µV)	% Height
1	12.702	3669708	94.85	99767	95.74
2	15.971	199243	5.15	4443	4.26

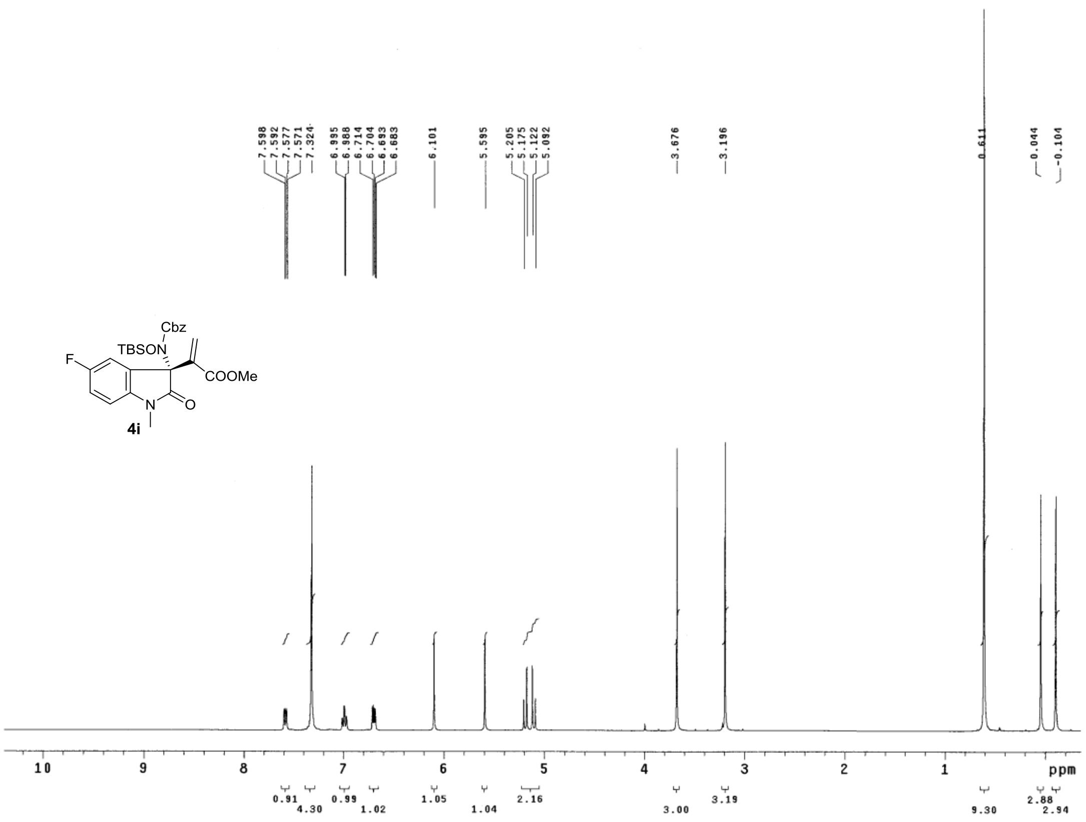
7.598
7.592
7.577
7.571
7.324
6.995
6.988
6.714
6.704
6.693
6.683
6.101
5.595
5.205
5.175
5.122
5.092
3.676
3.196
0.611
0.044
-0.104
Cbz
TBSON
F
COOMe
N
O
4i
10
9
8
7
6
5
4
3
2
1
ppm
0.91
4.30
0.99
1.02
1.05
1.04
2.16
3.00
3.19
9.30
2.88
2.94

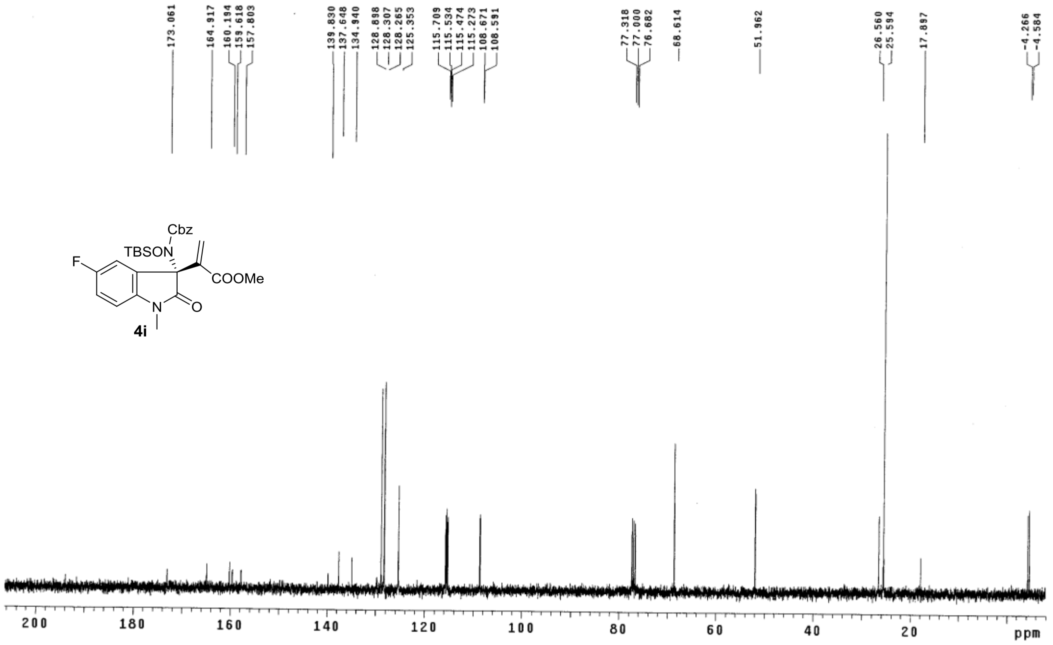
173.061
164.917
160.194
159.618
157.803
139.830
137.640
134.940
128.898
128.307
128.265
125.353
115.709
115.534
115.474
115.273
108.671
108.591
77.318
77.000
76.682
68.614
51.962
26.560
25.594
17.897
-4.266
-4.584
Cbz
TBSON
F
COOMe
N
O
4i
200
180
160
140
120
100
80
60
40
20
ppm

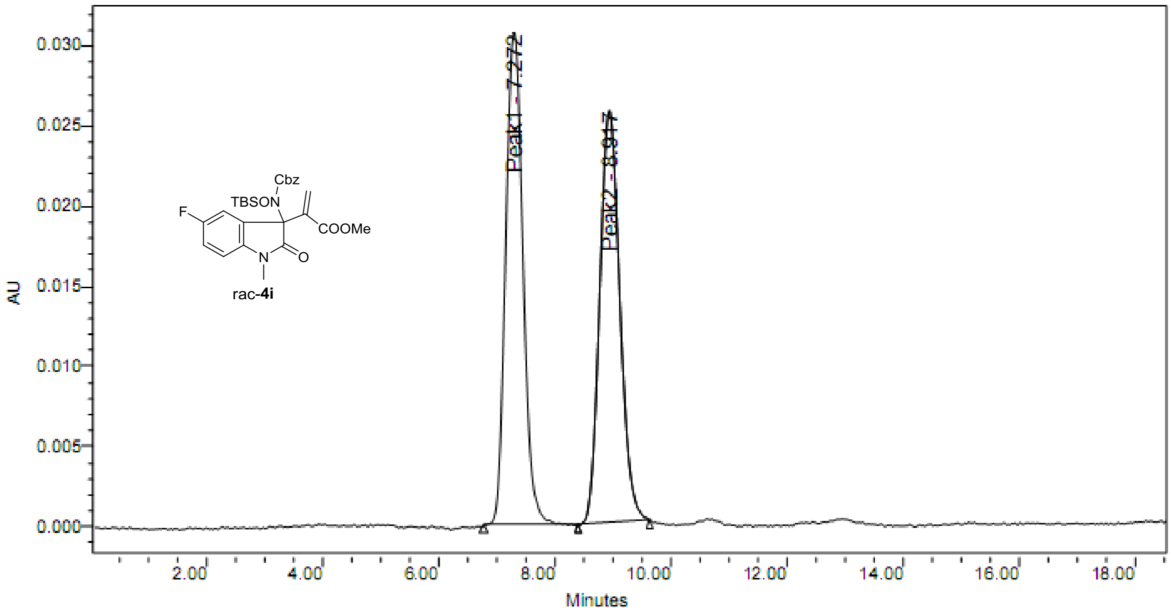

	Peak Name	RT (min)	Area (µV*sec)	% Area	Height (µV)	% Height
1	Peak1	7.272	641786	50.69	30809	54.47
2	Peak2	8.917	624374	49.31	25750	45.53

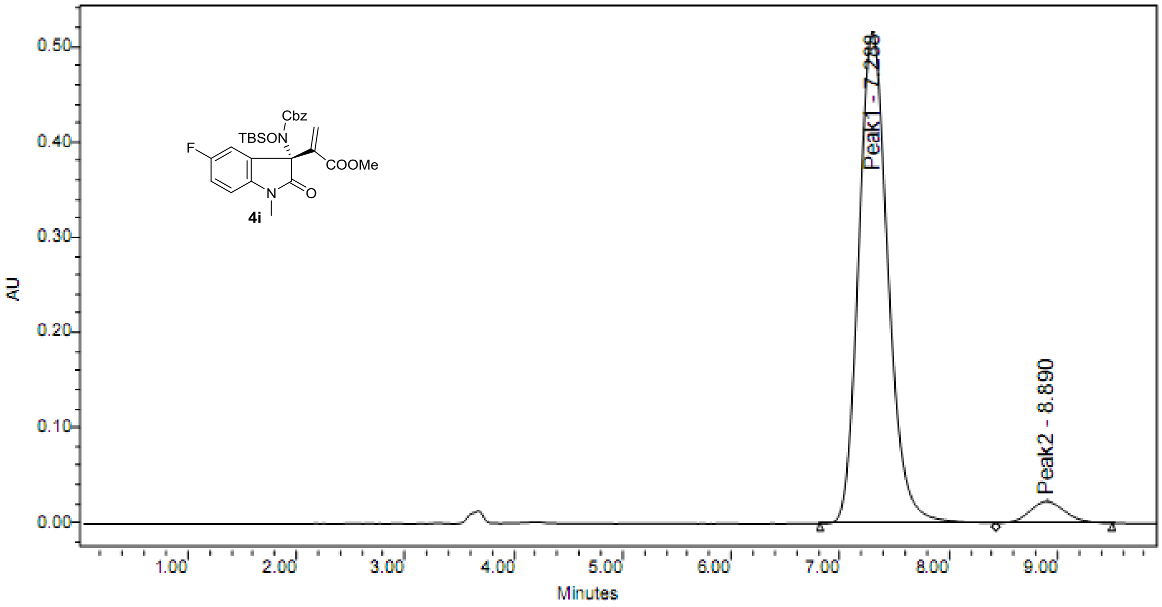

	Peak Name	RT (min)	Area (µV*sec)	% Area	Height (µV)	% Height
1	Peak1	7.288	9677512	94.90	516629	95.80
2	Peak2	8.890	519592	5.10	22676	4.20

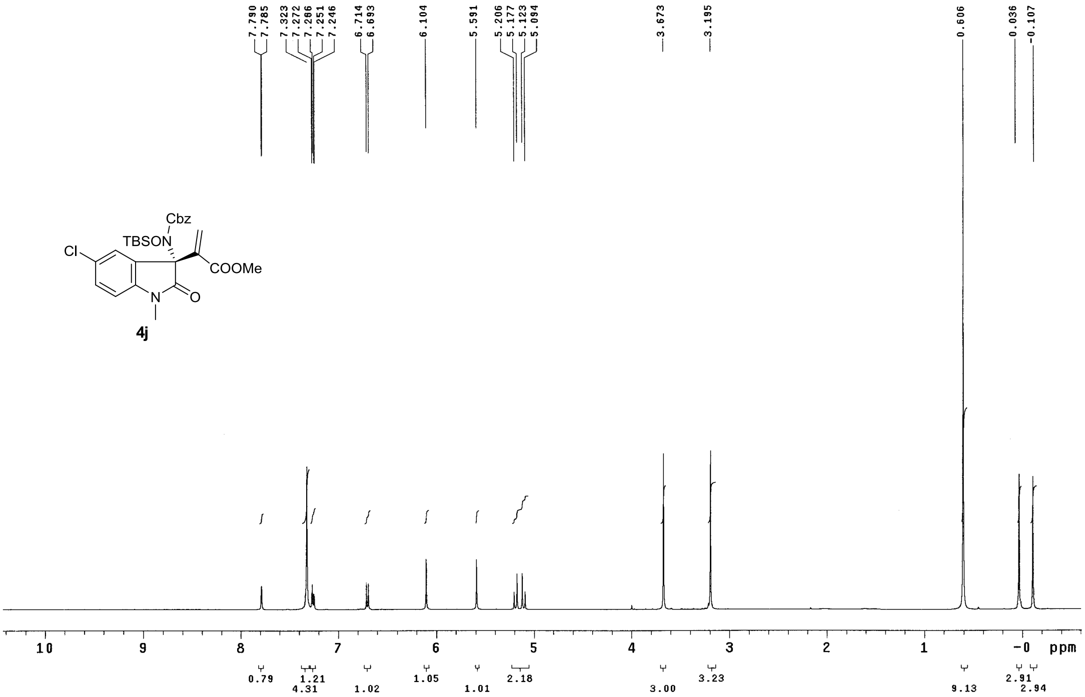
7.790
7.785
7.323
7.272
7.266
7.251
7.246
6.714
6.693
6.104
5.591
5.206
5.177
5.123
5.094
3.673
3.195
0.606
0.036
-0.107
Cbz
TBSON
Cl
COOMe
N
O
4j
0.79
1.21
4.31
1.02
1.05
1.01
2.18
3.00
3.23
9.13
2.91
2.94
ppm

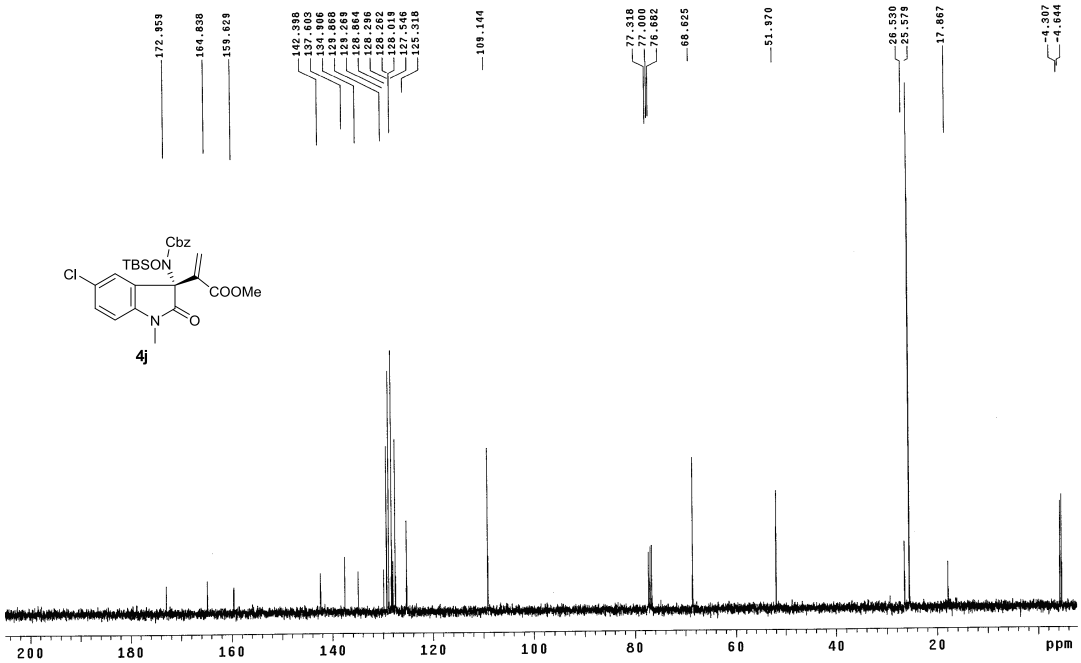
172.959
164.838
159.629
142.398
137.603
134.906
129.868
129.269
128.864
128.296
128.262
128.019
127.546
125.318
109.144
77.318
77.000
76.682
68.625
51.970
26.530
25.579
17.867
-4.307
-4.644
Cbz
TBSON
Cl
COOMe
N
O
4j
ppm

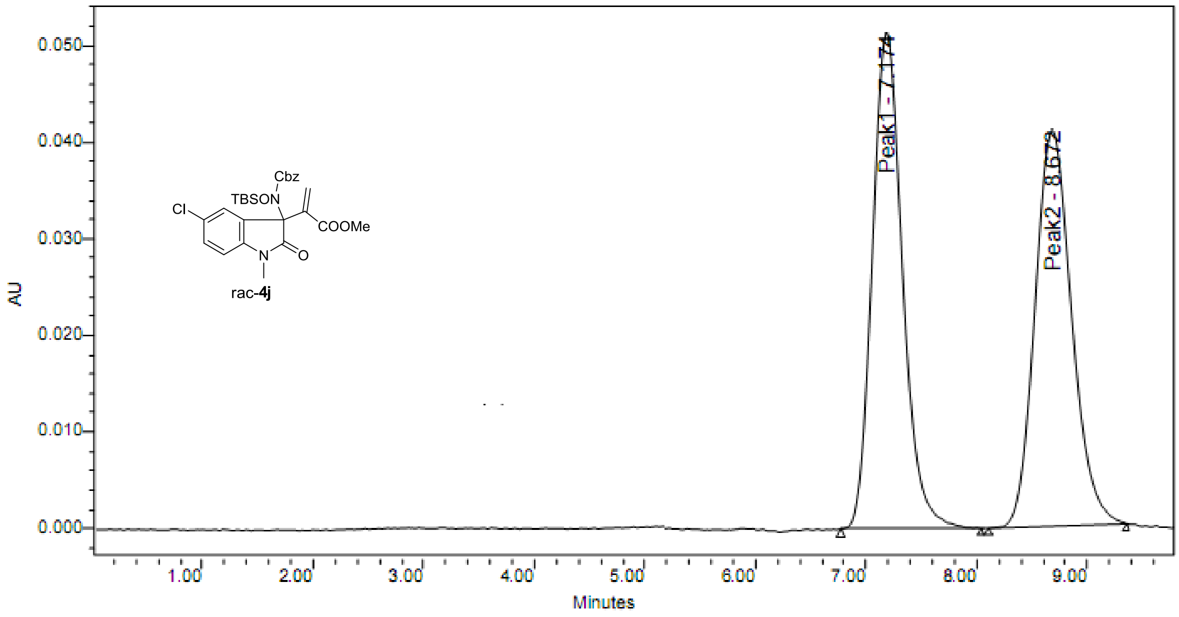

	Peak Name	RT (min)	Area (µV*sec)	% Area	Height (µV)	% Height
1	Peak1	7.174	975497	50.76	51401	55.65
2	Peak2	8.672	946167	49.24	40970	44.35

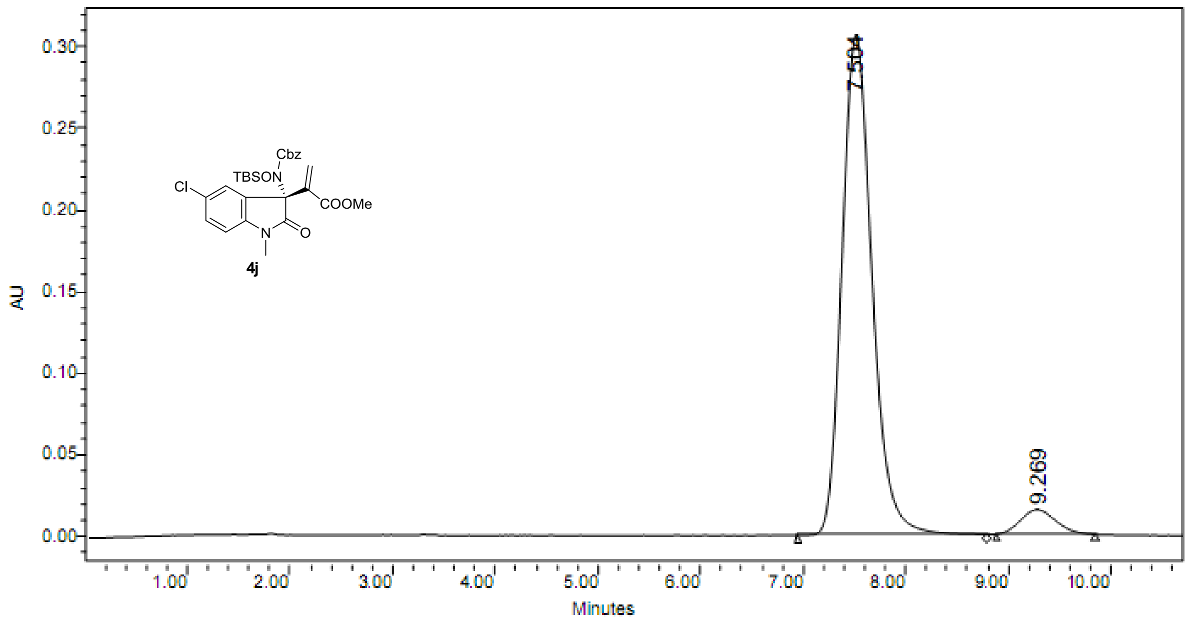

	RT (min)	Area (µV*sec)	% Area	Height (µV)	% Height
1	7.504	6252754	94.53	306410	95.31
2	9.269	361884	5.47	15089	4.69

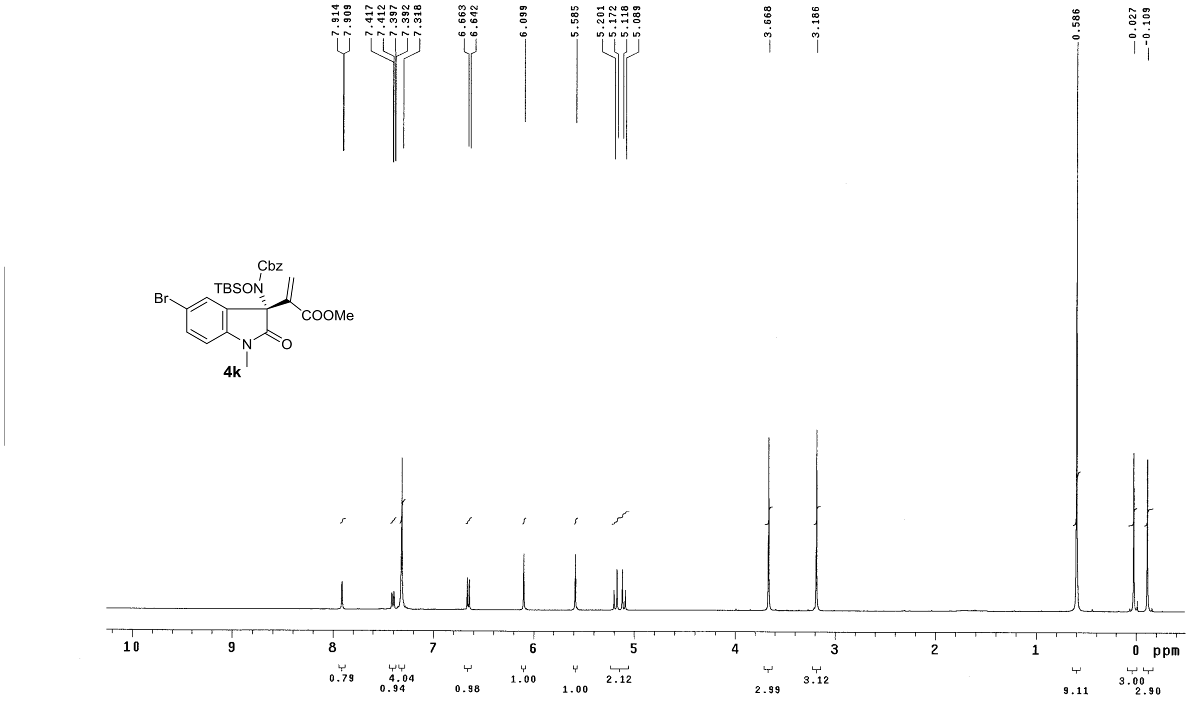

7.914
7.909
7.417
7.412
7.397
7.392
7.318
6.663
6.642
6.099
5.585
5.201
5.172
5.118
5.089
3.668
3.186
0.586
0.027
-0.109
Cbz
TBSON
Br
COOMe
N
O
4k
10 9 8 7 6 5 4 3 2 1 0 ppm
0.79
4.04
0.94
0.98
1.00
1.00
2.12
2.99
3.12
9.11
3.00
2.90

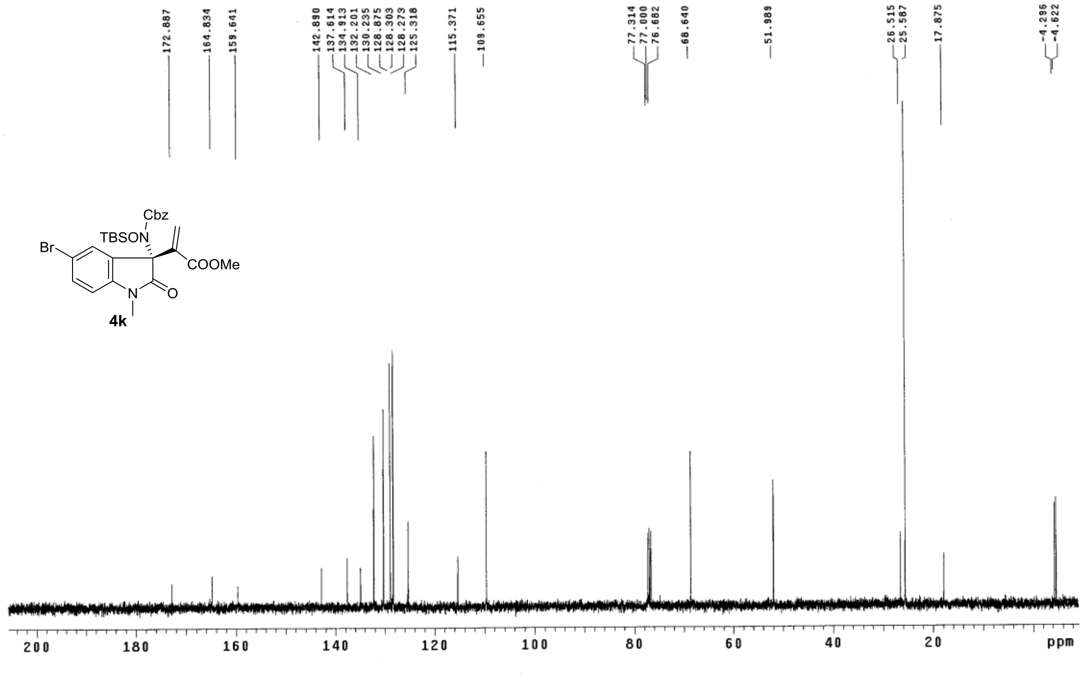

172.887
164.834
159.641
142.890
137.614
134.913
132.201
130.295
128.875
128.303
128.273
125.318
115.371
109.655
77.314
77.000
76.682
68.640
51.988
26.515
25.587
17.875
-4.296
-4.622
Cbz
TBSON
Br
COOMe
N
O
4k
200 180 160 140 120 100 80 60 40 20 ppm

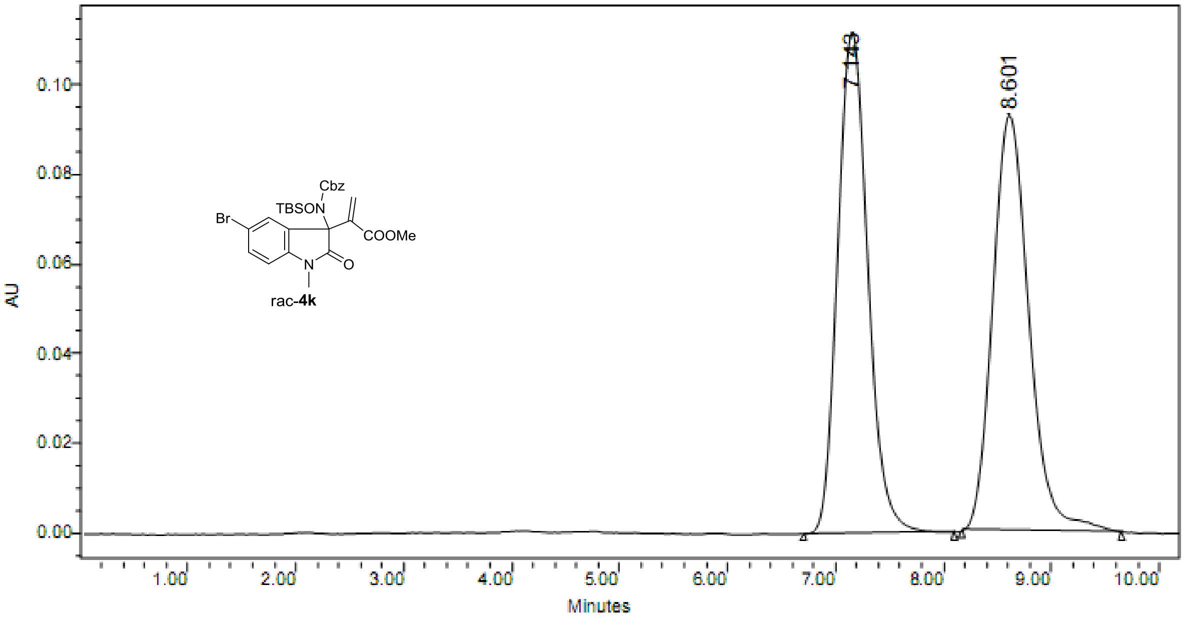

	RT (min)	Area (µV*sec)	% Area	Height (µV)	% Height
1	7.143	2177297	50.08	111955	54.68
2	8.601	2170090	49.92	92795	45.32

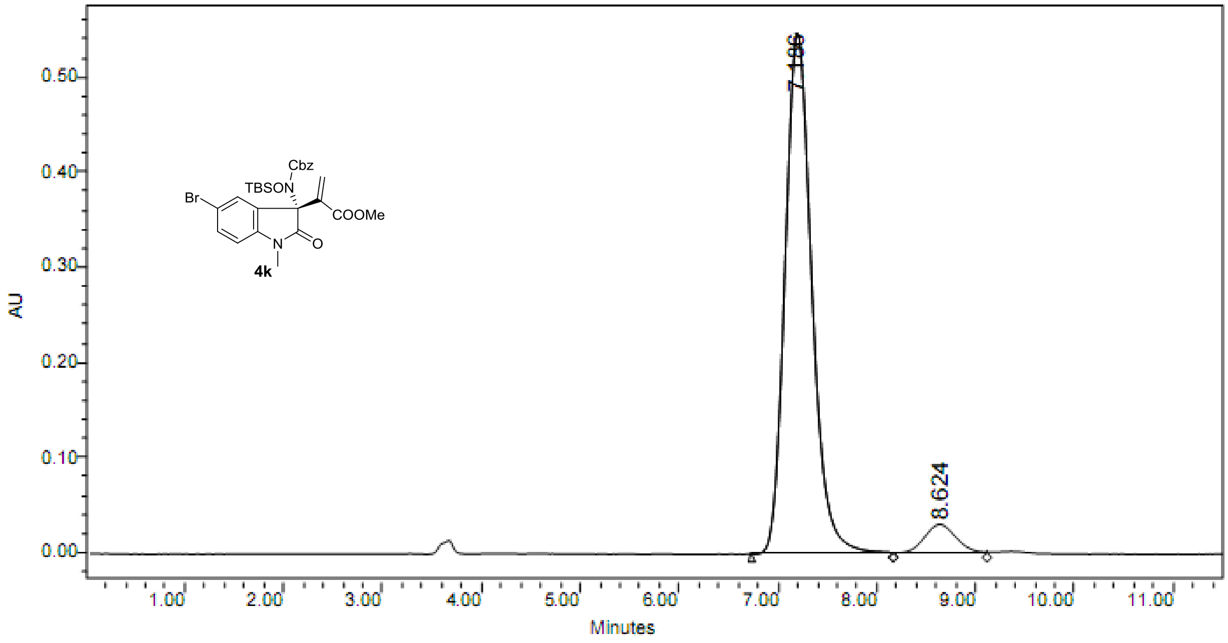

	RT (min)	Area (µV*sec)	% Area	Height (µV)	% Height
1	7.186	10347245	93.93	547314	94.62
2	8.624	727605	6.07	31110	5.38

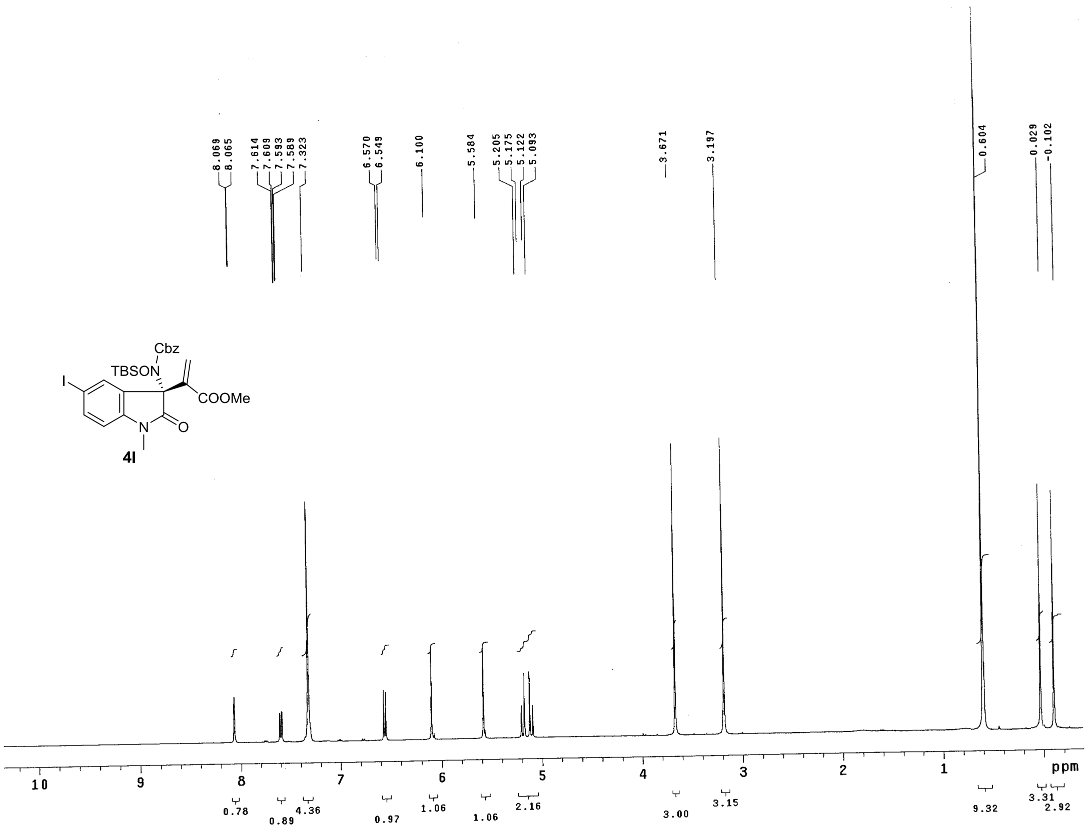
8.069
8.065
7.614
7.609
7.593
7.589
7.323
6.570
6.549
6.100
5.584
5.205
5.175
5.122
5.093
3.671
3.197
0.604
0.029
-0.102
Cbz
TBSON
I
COOMe
N
O
4l
10 9 8 7 6 5 4 3 2 1 ppm
0.78 0.89 4.36 0.97 1.06 1.06 2.16 3.00 3.15 9.32 3.31 2.92

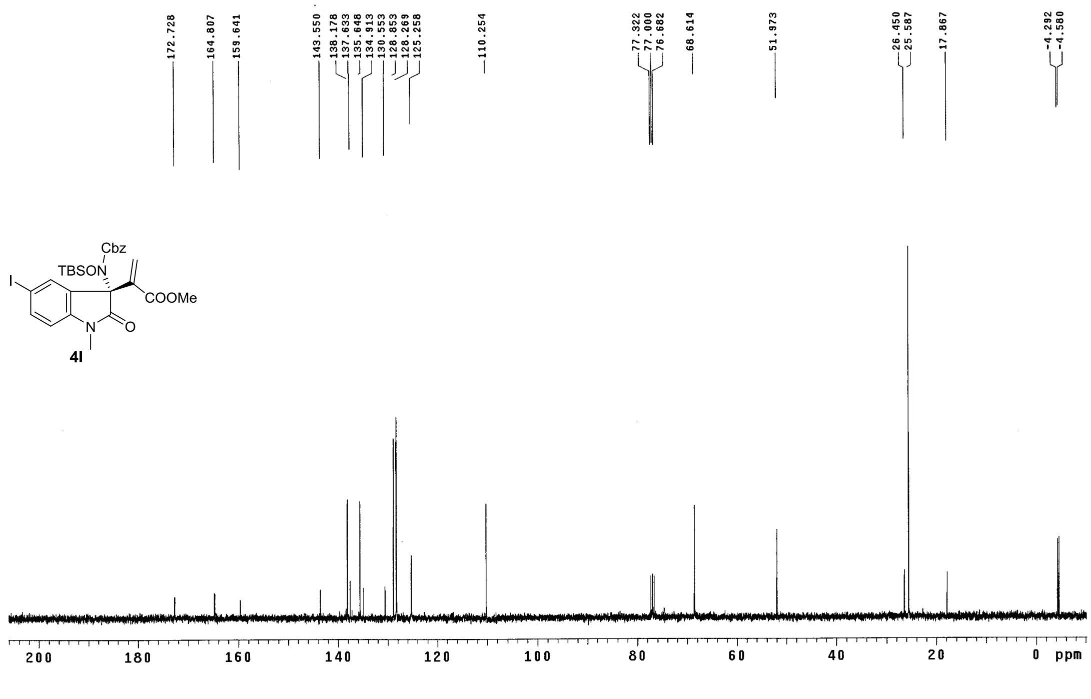
172.728
164.807
159.641
143.550
138.178
137.633
135.648
134.913
130.553
128.853
128.269
125.258
110.254
77.322
77.000
76.682
68.614
51.973
26.450
25.587
17.867
-4.292
-4.580
Cbz
TBSON
I
COOMe
N
O
4l
200 180 160 140 120 100 80 60 40 20 0 ppm

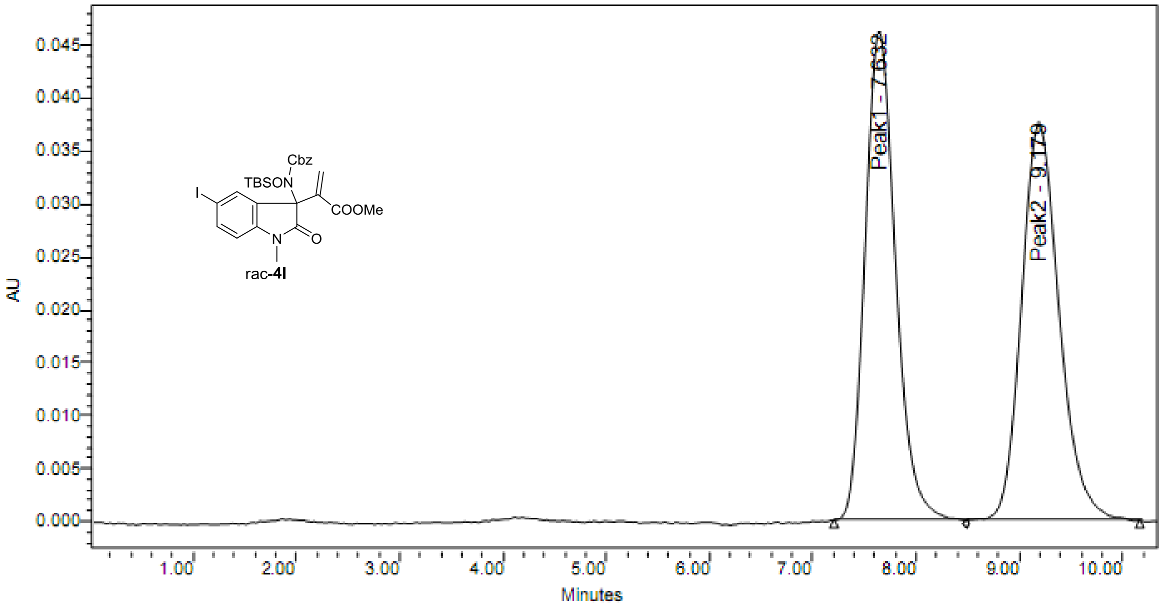

	Peak Name	RT (min)	Area (µV*sec)	% Area	Height (µV)	% Height
1	Peak1	7.632	950992	49.85	46183	55.20
2	Peak2	9.179	956900	50.15	37476	44.80

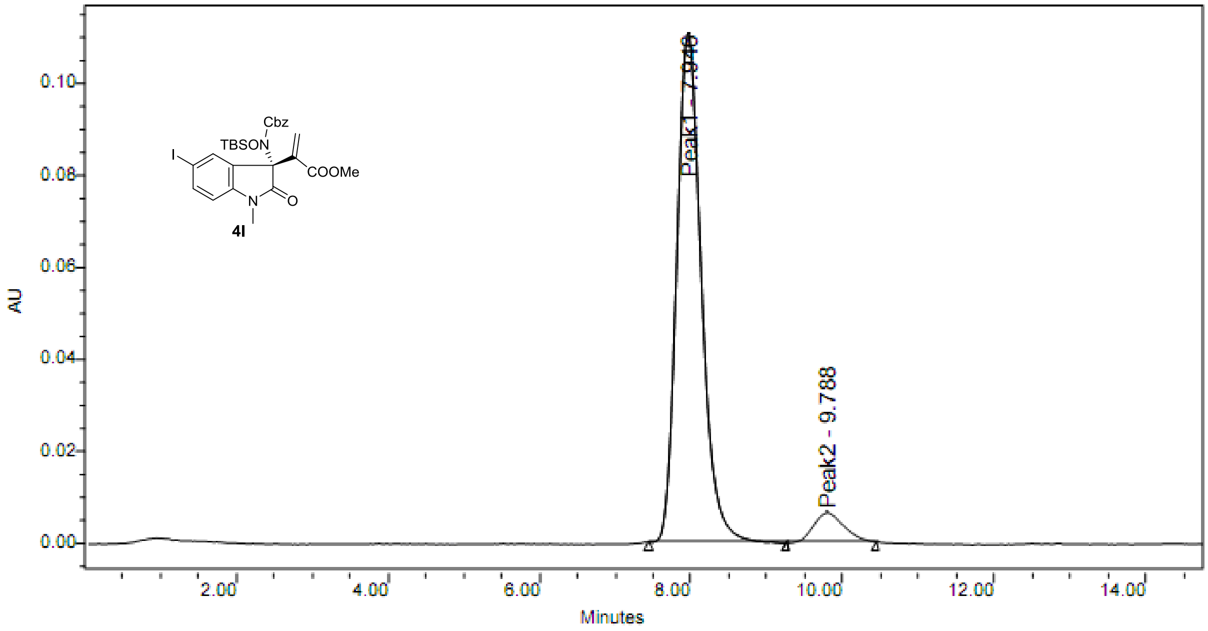

	Peak Name	RT (min)	Area (µV*sec)	% Area	Height (µV)	% Height
1	Peak1	7.946	2498444	93.10	110805	94.49
2	Peak2	9.788	185210	6.90	6460	5.51

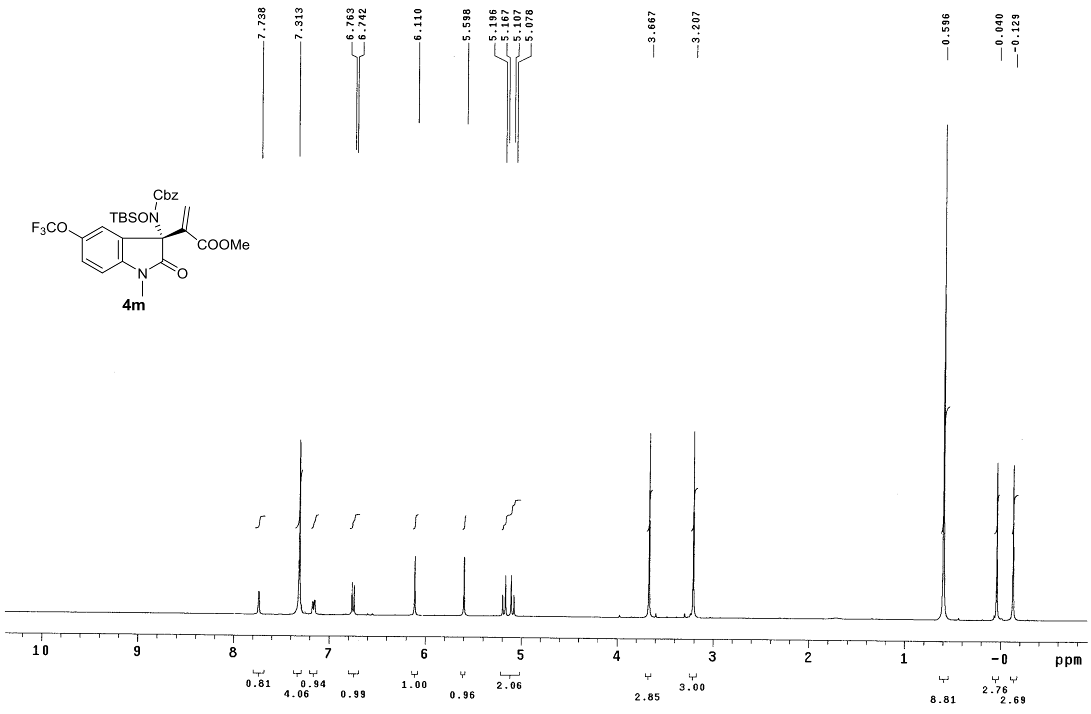
7.738
7.313
6.763
6.742
6.110
5.598
5.196
5.167
5.107
5.078
3.667
3.207
0.596
0.040
-0.129
Cbz
TBSON
F3CO
COOMe
N
O
4m
10 9 8 7 6 5 4 3 2 1 -0 ppm
0.81 4.06 0.94 0.99 1.00 0.96 2.06 2.85 3.00 8.81 2.76 2.69

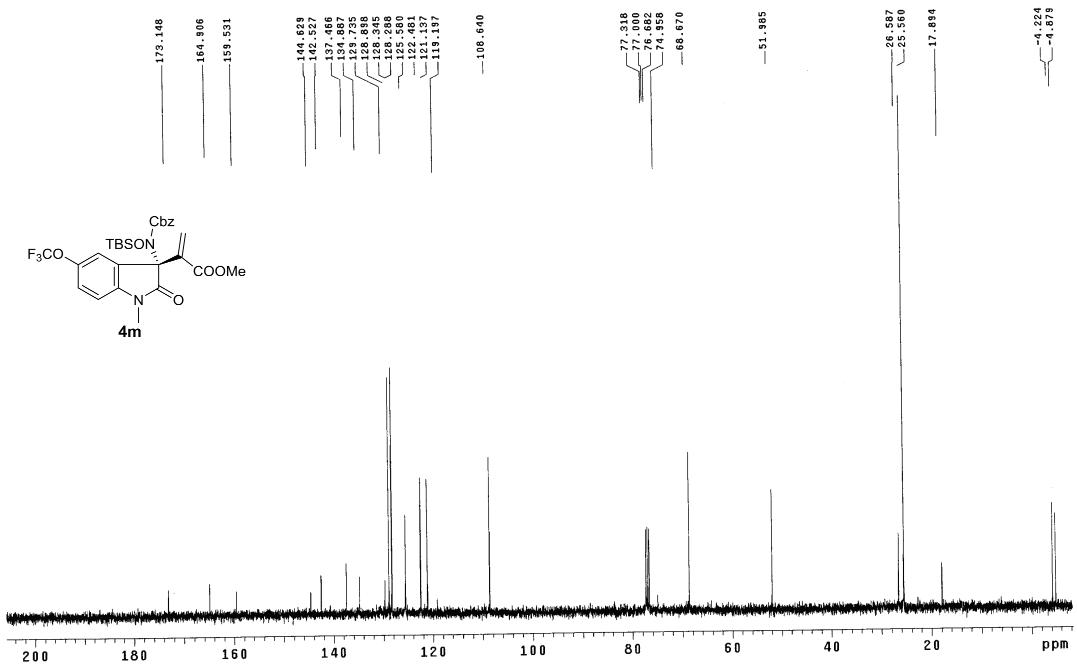
173.148
164.906
159.531
144.629
142.527
137.466
134.887
129.735
128.898
128.345
128.288
125.580
122.481
121.137
119.197
108.640
77.318
77.000
76.682
74.958
68.670
51.985
26.587
25.560
17.894
-4.224
-4.879
Cbz
TBSON
F3CO
COOMe
N
O
4m
200 180 160 140 120 100 80 60 40 20 ppm

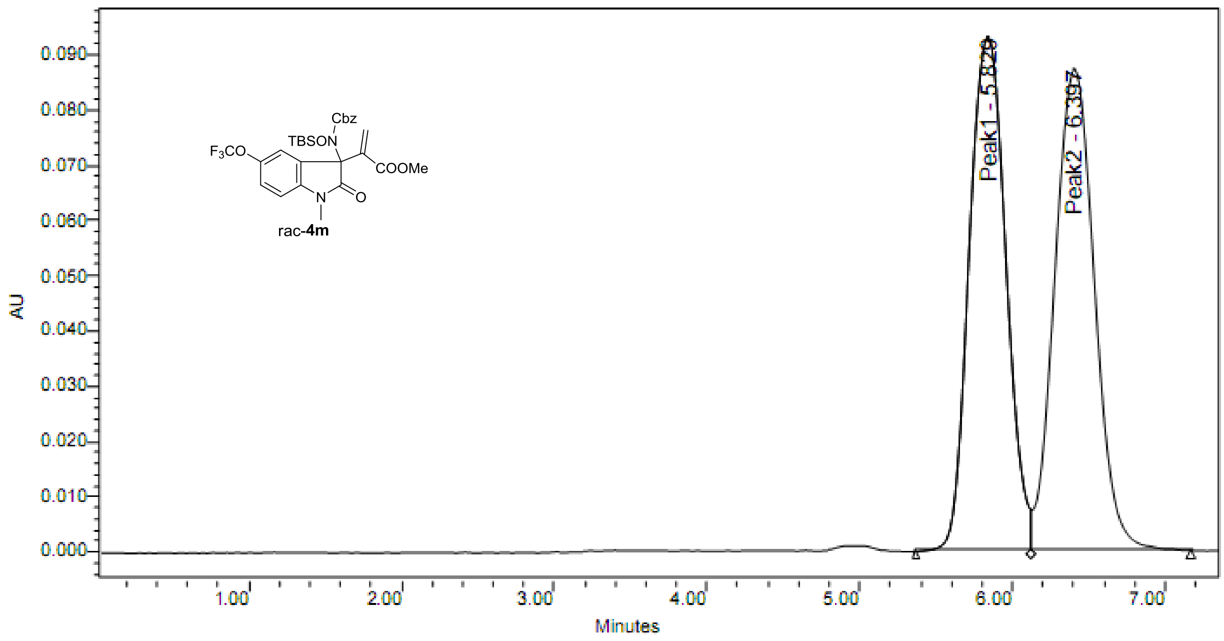

	Peak Name	RT (min)	Area (µV*sec)	% Area	Height (µV)	% Height
1	Peak1	5.829	1562003	49.58	93275	51.65
2	Peak2	6.397	1588384	50.42	87309	48.35

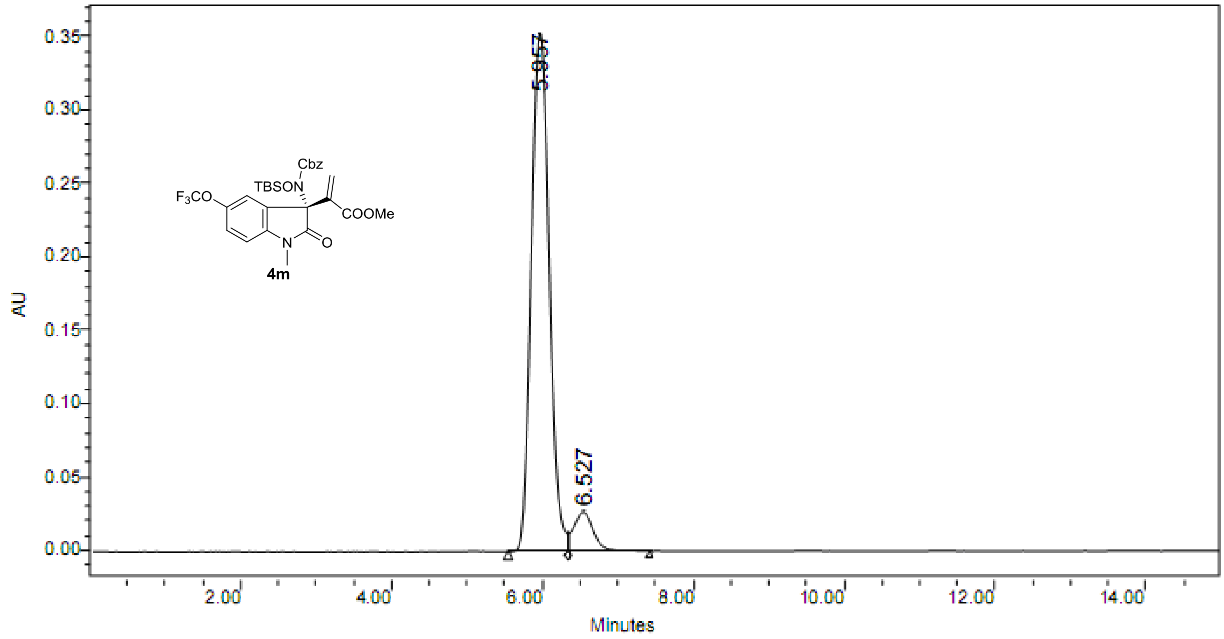

	RT (min)	Area (µV*sec)	% Area	Height (µV)	% Height
1	5.957	5838881	92.30	353402	93.05
2	6.527	486900	7.70	26398	6.95

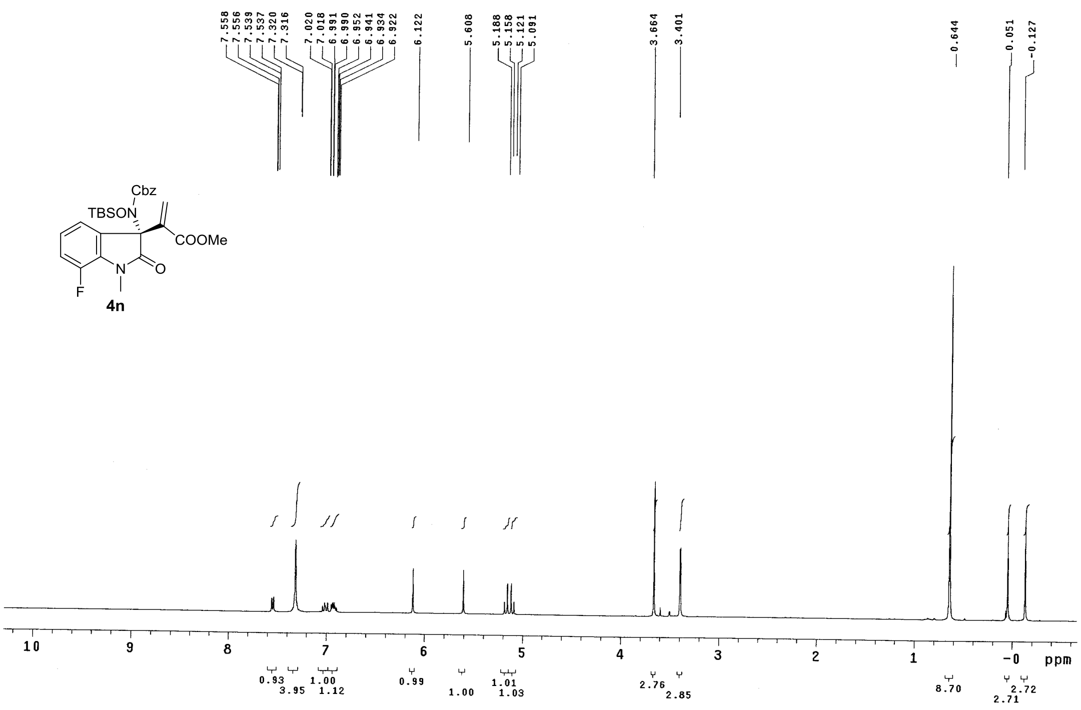
7.558
7.556
7.539
7.537
7.320
7.316
7.020
7.018
6.991
6.990
6.952
6.941
6.934
6.922
6.122
5.608
5.188
5.158
5.121
5.091
3.664
3.401
0.644
0.051
-0.127
Cbz
TBSON
COOMe
N
O
F
4n
10 9 8 7 6 5 4 3 2 1 -0 ppm
0.93
3.95
1.00
1.12
0.99
1.00
1.01
1.03
2.76
2.85
8.70
2.71
2.72

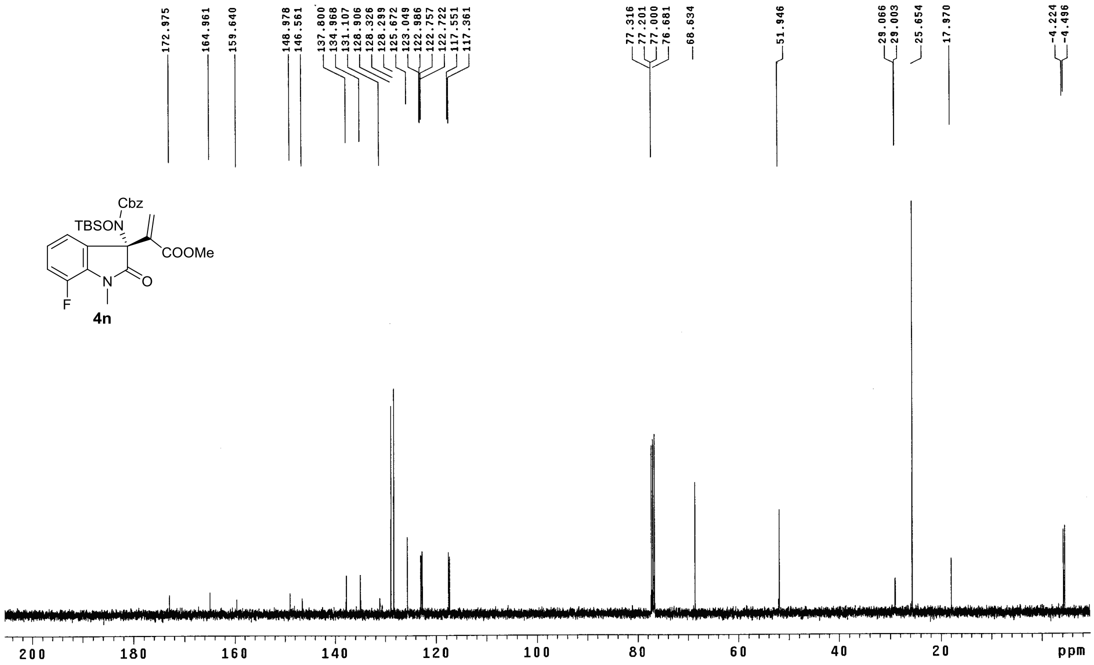
172.975
164.961
159.640
148.978
146.561
137.800
134.968
131.107
128.906
128.326
128.299
125.672
123.049
122.986
122.757
122.722
117.551
117.361
77.316
77.201
77.000
76.681
68.634
51.946
29.066
29.003
25.654
17.970
-4.224
-4.496
Cbz
TBSON
COOMe
N
O
F
4n
200 180 160 140 120 100 80 60 40 20 ppm

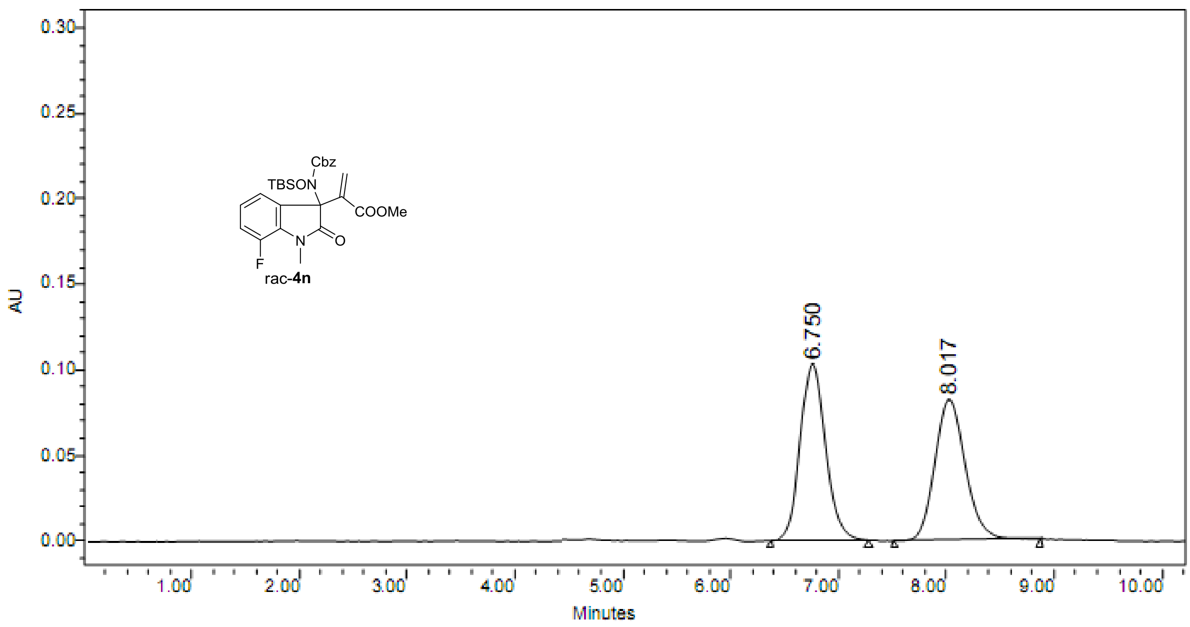

	RT (min)	Area (µV*sec)	% Area	Height (µV)	% Height
1	6.750	1708260	51.24	103140	55.66
2	8.017	1625556	48.76	82170	44.34

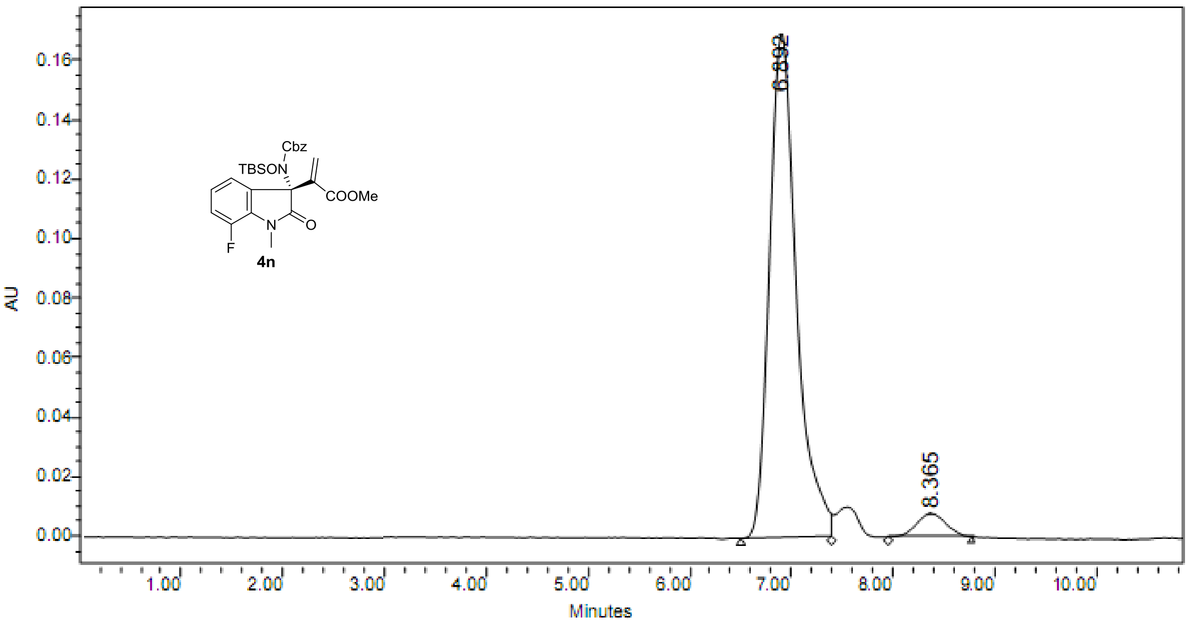

	RT (min)	Area (µV*sec)	% Area	Height (µV)	% Height
1	6.892	3143933	95.18	169780	95.60
2	8.365	159382	4.82	7811	4.40

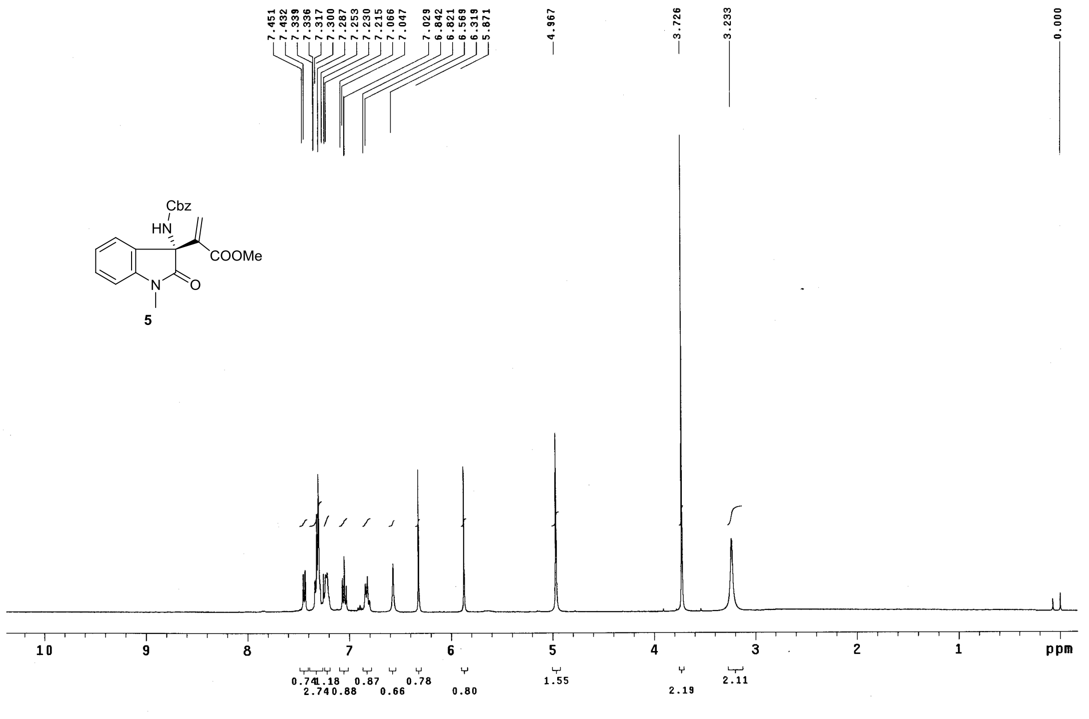
7.451
7.432
7.339
7.336
7.317
7.300
7.287
7.253
7.230
7.215
7.066
7.047
7.029
6.842
6.821
6.569
6.319
5.871
4.967
3.726
3.233
0.000
Cbz
HN
COOMe
N
O
5
ppm
0.74
1.18
0.87
0.78
2.74
0.88
0.66
0.80
1.55
2.19
2.11

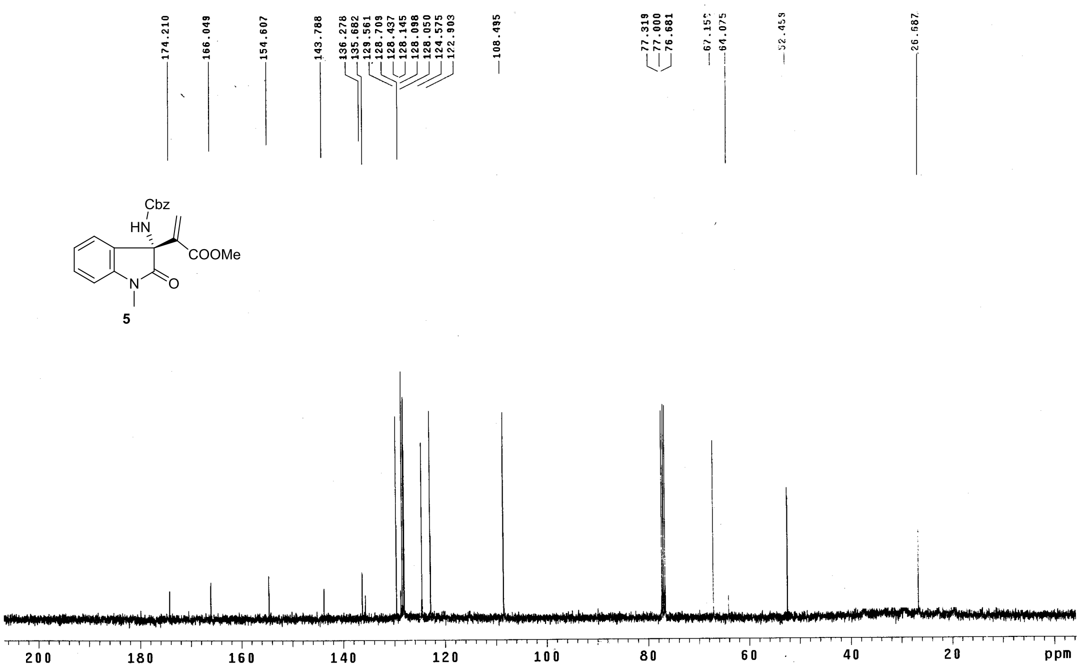
174.210
166.049
154.607
143.788
136.278
135.682
129.561
128.709
128.437
128.145
128.098
128.050
124.575
122.903
108.495
77.319
77.000
76.681
26.687
Cbz
HN
COOMe
N
O
5
ppm

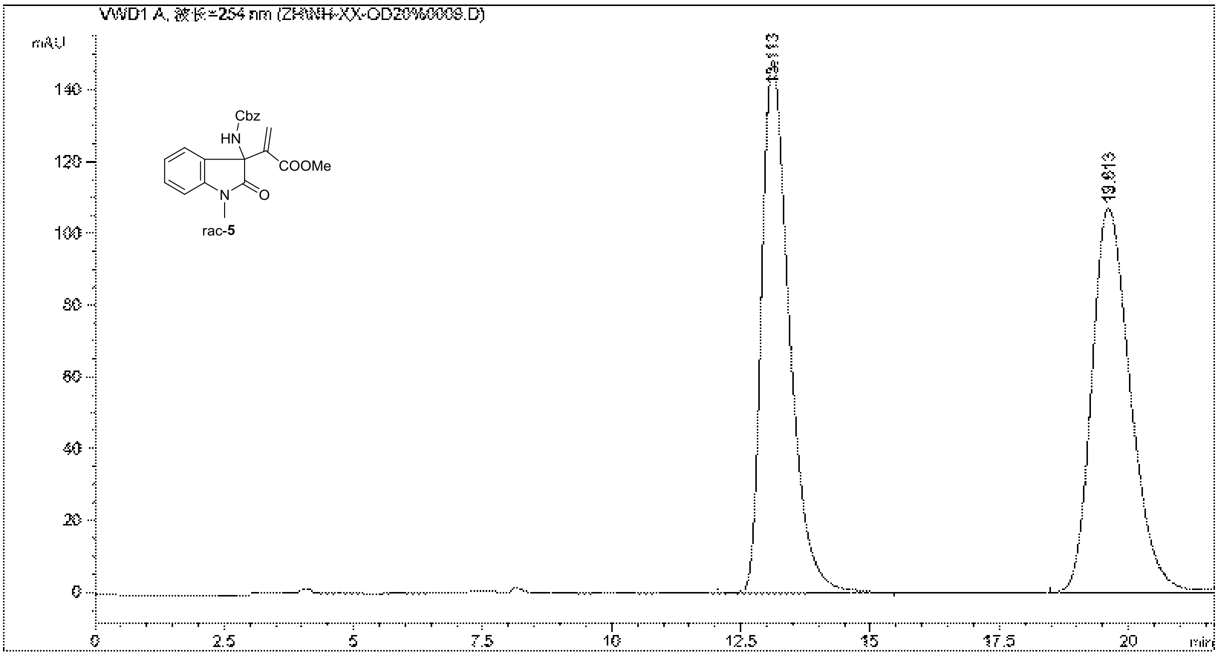

#	[min]		[min]	mAU *s	[mAU]	%
1	13.113	VB	0.5677	5527.01123	148.65058	49.8333
2	19.613	BB	0.8043	5563.98584	107.06664	50.1667

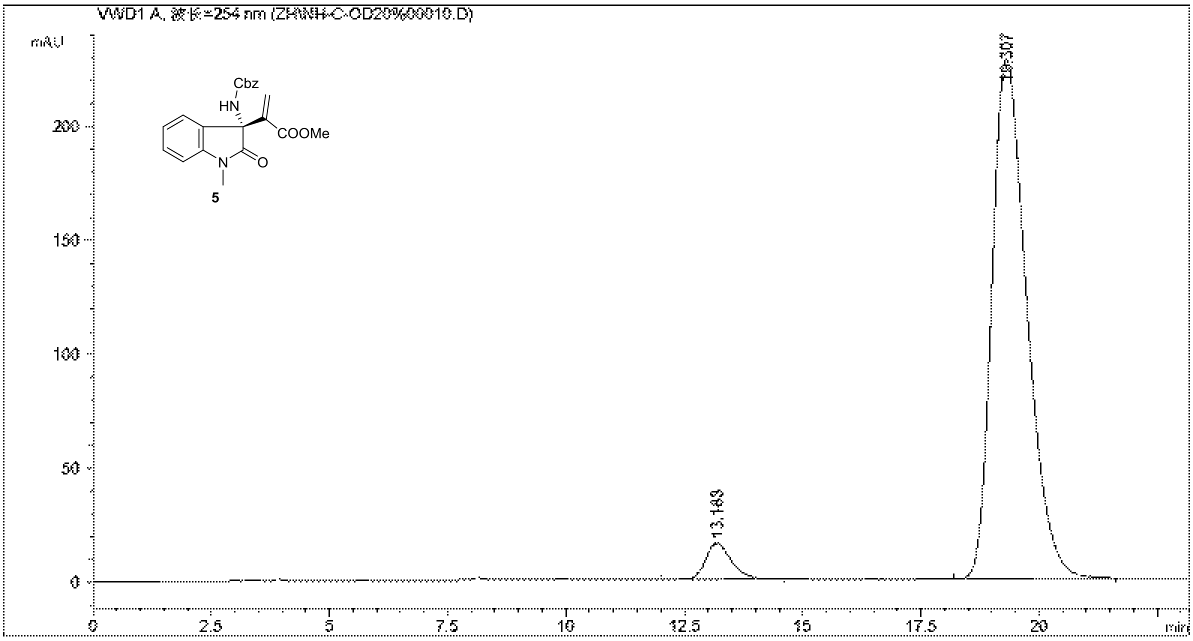

#	[min]		[min]	mAU *s	[mAU]	%
1	13.183	VB	0.5804	604.95105	15.80855	4.9305
2	19.307	BB	0.7887	1.16646e4	228.14536	95.0695

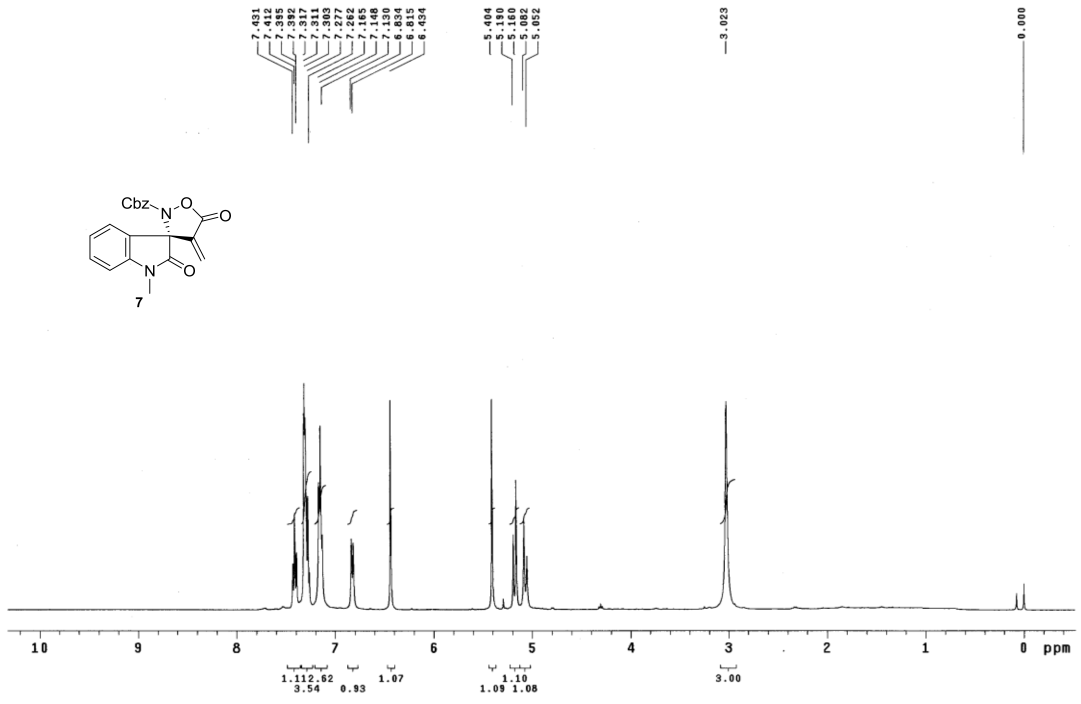
7.431
7.412
7.395
7.392
7.317
7.311
7.303
7.277
7.262
7.165
7.148
7.130
6.834
6.815
6.434
5.404
5.190
5.160
5.082
5.052
3.023
0.000
Cbz
N
O
7
ppm

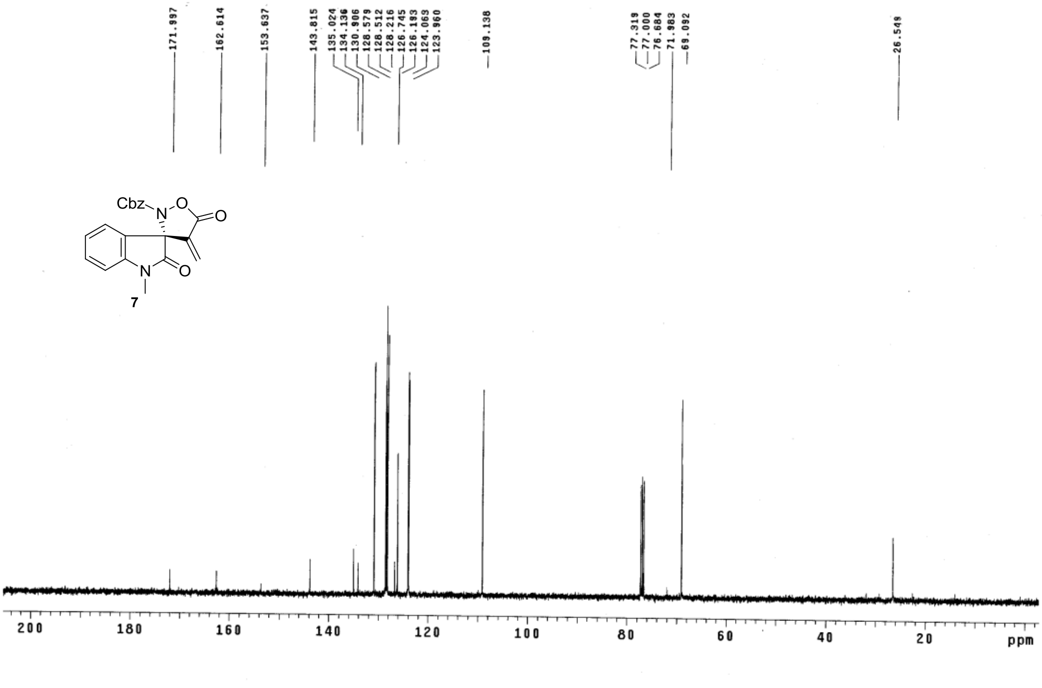
171.997
162.614
153.637
143.815
135.024
134.136
130.906
128.579
128.512
128.216
126.745
126.193
124.063
123.900
109.138
77.319
77.000
76.684
71.983
69.092
26.549
Cbz
N
O
7
ppm

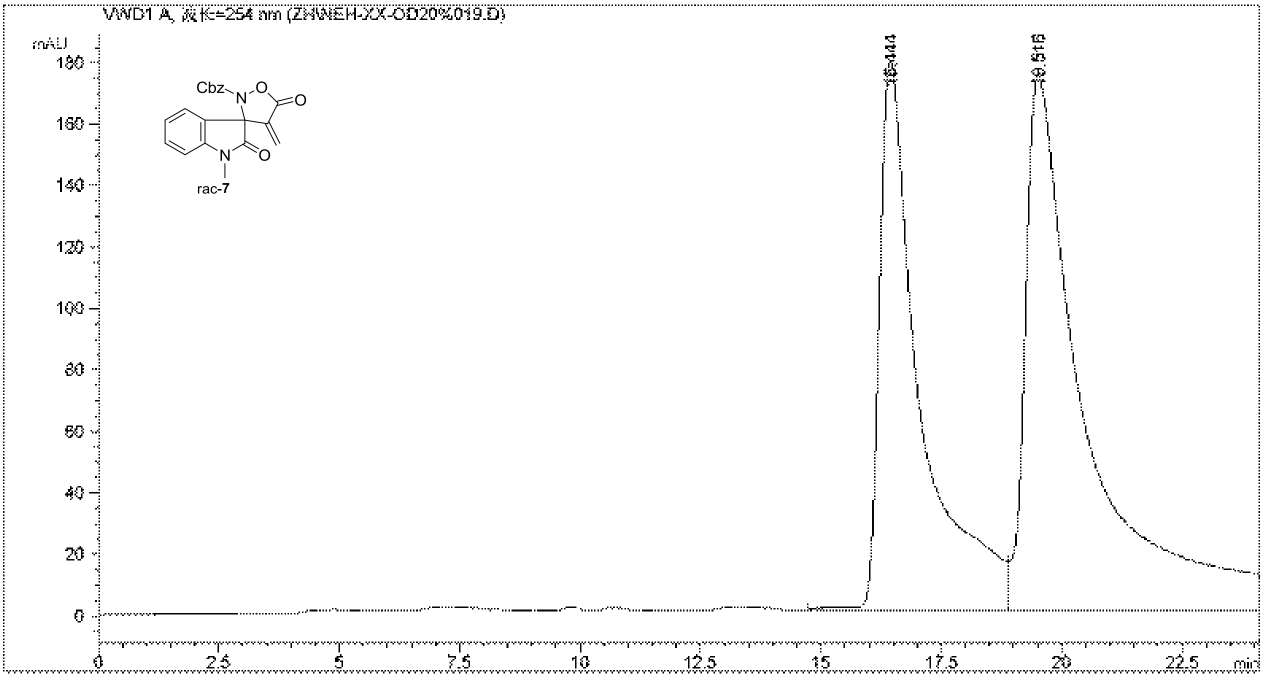

#	[min]		[min]	mAU *s	[mAU]	%
1	16.444	BV	0.8291	1.04819e4	178.53511	46.4217
2	19.516	VBA	1.1734	1.46234e4	173.92662	53.5783

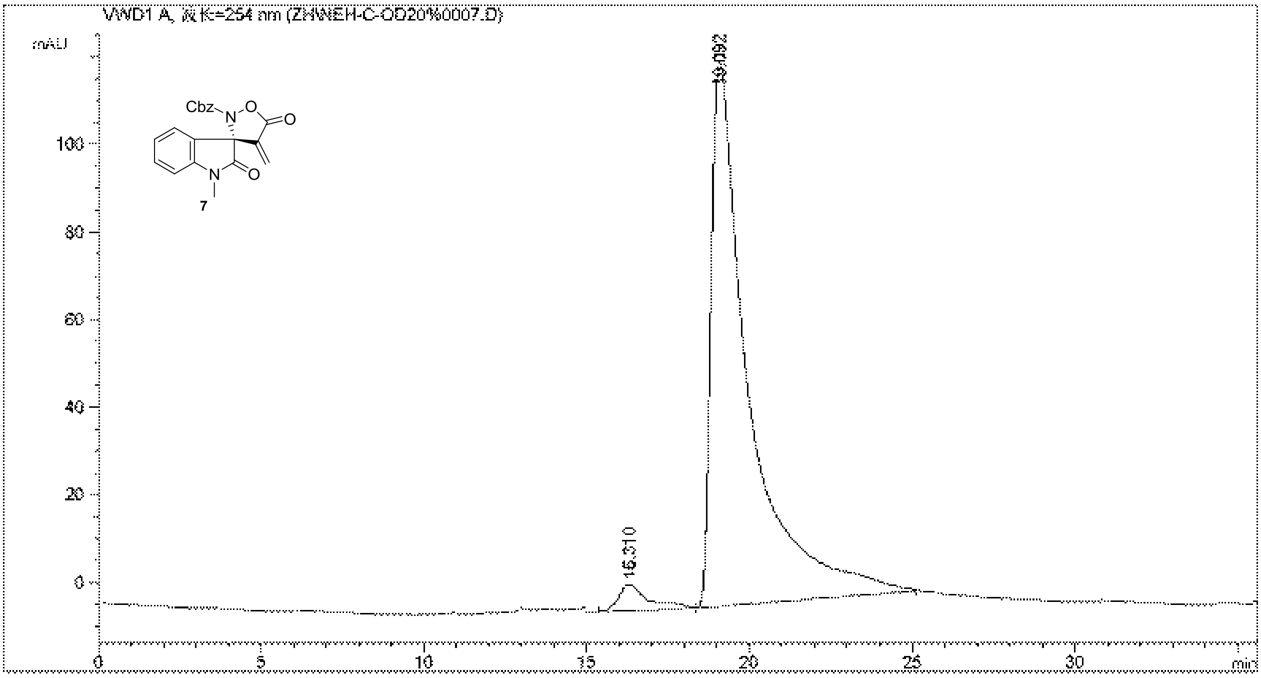

#	[min]		[min]	mAU *s	[mAU]	%
1	16.310	BB	0.8939	379.64362	5.96525	5.1383
2	19.092	BB	1.1322	1.00549e4	124.21668	94.8617

7. References

1. Waldmann, H.; Khedkar, V.; Dückert, H.; Schürmann, M.; Oppel, I.-M.; Kumar, K. *Angew. Chem. Int. Ed.* **2008,** *47*, 6869–6872.

2. Peng, J.; Huang, X.; Jiang, L.; Cui, H. L.; Chen, Y. C. *Org. Lett.* **2011,** *13,* 4584–4587.

3. Chung, Y. M.; Im, Y. J.; Kim, J. N. *Bull. Korean Chem. Soc.* **2002,** *23,* 1651–1654.

4. Chen, Y. K.; Yoshida, M.; MacMillan, D. W. C. *J. Am. Chem. Soc.* **2006,** *128,* 9328–9329.
